# Mechanism of Solid Solution‐Driven Texture Induced by Ag Doping in YBCO Superconductor

**DOI:** 10.1002/advs.202522923

**Published:** 2026-02-10

**Authors:** Fenyan Zhao, Baoqiang Zhang, Xiyang Su, Yantang Zhao, Xingyi Zhang

**Affiliations:** ^1^ Key Laboratory of Mechanics on Disaster and Environment in Western China attached to the Ministry of Education of China Lanzhou University Lanzhou Gansu P. R. China; ^2^ Department of Mechanics and Engineering Sciences College of Civil Engineering and Mechanics Lanzhou University Lanzhou Gansu P. R. China; ^3^ Department of Mechanical and Aerospace Engineering The Hong Kong University of Science and Technology Hong Kong P. R. China

**Keywords:** Ag‐doping, coherent interface, HTS, solid solution‐driven texture, YBCO

## Abstract

Since the discovery of YBa_2_Cu_3_O_7‐δ_ (YBCO or Y123), enhancing its superconducting and mechanical properties has been a major focus. Ag doping is a promising strategy for bulk materials, but its mechanism remains debated, particularly regarding whether Ag segregates at grain boundaries or enters the lattice. The origin of the enhancement remains unclear, as conventional powder‐doping methods hinder gradient formation and obscure key effects at specific doping levels. Here, we propose a dual‐material co‐extrusion and freeze‐drying strategy to construct a macroscopic Ag‐YBCO interface, where directional pores in YBCO enable Ag diffusion and compositional gradient formation at high temperatures. Experiments reveal a [001]‐oriented YBa_2_Cu_3‐x_Ag_x_O_7‐δ_ solid solution with coherent interfaces with Y123, which transmit crystallographic orientation at the atomic scale and promote orientated Y123 growth. First‐principles calculations reveal that Ag substitution‐induced lattice relaxation plays a key role in driving texture formation. As a result, the composite exhibits a significantly enhanced critical current density (*J*_c_) while maintaining a stable critical temperature (*T*_c_), accompanied by a transition in the fracture behavior from intergranular to transgranular fracture. This work reveals the mechanism of solid solution‐driven texture induced by Ag doping in YBCO, providing new insights into dopant‐mediated texture evolution in REBCO (Re = rare earth) superconductors.

## Graphical abstract

Ag‐doped YBCO with a designed concentration gradient is produced by dual‐material co‐extrusion and freeze‐drying. During annealing, Ag^+^ ions diffuse into Y123, substituting for Cu(1) and inducing *c*‐axis relaxation. This leads to the formation of [001]‐oriented YBCAO, creating a coherent interface that templates the textured growth of Y123. Consequently, the *J*_c_ at 77 K is significantly enhanced, and the fracture mode transitions to transgranular.

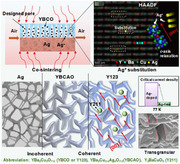

## 1 Introduction

Since its discovery in the late 1980s [1, 2], the YBCO superconductor has attracted sustained research interest due to its *T*_c_ exceeding the boiling point of liquid nitrogen (77 K). Studies have focused on optimizing its properties [3], elucidating its structural characteristics [4], and exploring practical applications in high‐field magnets, energy transmission, and electronic devices [5, 6]. Nevertheless, its intrinsic brittleness, characteristic of a ceramic material, compromises its processability and structural stability [7]. Furthermore, its perovskite‐type layered structure renders the superconducting properties highly dependent on electron transport within the CuO_2_ planes [8]. To address these materials science challenges, chemical modification and regulation of the YBCO has emerged as a central strategy. Consequently, elemental doping has received widespread attention as a potential approach to enhance the performance of YBCO. Previous studies have demonstrated that different dopants can optimize the performance of YBCO through distinct mechanisms. For instance, CeO_2_ doping in bulk YBCO promotes the refinement of Y_2_BaCuO_5_ (Y211) particles, thereby enhancing flux pinning [9], while substitution of non‐magnetic Y^3+^ with magnetic RE ions (Nd, Sm, Gd, Dy, etc.) introduces localized magnetic moments that serve as additional pinning centers for flux vortices [10].

Bell Laboratories first demonstrated the compatibility of silver as a substrate for YBCO [11]. Since then, the role and interaction mechanisms of Ag in the YBCO system have attracted sustained attention, with research evolving to include its mechanical properties, superconducting performance, and lattice structure [12, 13, 14, 15, 16, 17, 18, 19, 20, 21, 22, 23, 24, 25, 26, 27, 28, 29, 30, 31, 32, 33, 34, 35]. Existing studies have demonstrated that the addition of Ag into YBCO does not compromise its superconducting properties. Furthermore, it significantly enhances *J*_c_ by strengthening flux pinning effects [12, 14, 15, 19, 22, 24, 25, 27, 30, 32, 33]. Moreover, the incorporation of Ag markedly improves the mechanical properties of the YBCO system, particularly in terms of fracture toughness and bending strength [14, 22, 34]. Additionally, it has been reported that a specific Ag doping level can induce texture in YBCO, although the underlying mechanism remains unclear [24, 32, 33, 35]. However, the doping mechanism of Ag in the YBCO system remains controversial in academia, primarily focusing on whether Ag exists as grain boundary precipitates or forms a solid solution by substituting atoms in the YBCO lattice [12, 13, 15, 20, 21, 32]. It is widely accepted that the segregation of a small amount of Ag particles at grain boundaries facilitates the bridging of superconducting grains, thereby enhancing both the superconductivity and mechanical properties of the material [14, 15, 22, 24, 27, 28, 29, 33, 35]. When Ag^+^ substitutes for Cu^2+^ at the Cu(1) sites in the Cu‐O chains, forming a substitutional solid solution YBa_2_Cu_3‐x_Ag_x_O_7‐δ_ (YBCAO), low levels of Ag^+^ substitution do not significantly affect *T*_c_ [12, 20, 21, 32, 35]. However, excessive Ag doping can lead to an increase in oxygen vacancies to maintain charge neutrality, which in turn results in a degradation of superconducting performance [13, 21, 24]. Yet it remains unclear whether the YBCAO phase is spatially homogeneous, whether its presence affects the crystallographic orientation or grain size of the Y123 superconducting phase, and what the underlying mechanisms of such effects might be.

In the field of material doping modification, the thermal diffusion method has been widely adopted as a classical and conventional approach, owing to its advantages such as low cost, simple processing, and minimal equipment requirements. Typically, this technique involves directly mixing the host material with dopant powders and then sintering the mixture at high temperatures. During this process, atomic‐scale solid‐state diffusion allows dopant atoms to incorporate into the host lattice [36, 37]. Owing to these features, this technique has been extensively employed in studies of the Ag‐YBCO system [12, 13, 15, 16, 21, 22, 24, 27, 28, 29, 31, 33, 35]. However, samples prepared using this method exhibit a uniform dopant distribution and cannot form a concentration gradient, making it difficult to observe the dynamic interactions and evolution between the dopant and the host material at different sintering stages. To overcome this limitation, researchers have been actively developing approaches to through pre‐designed material architectures [38, 39]. Previous studies have demonstrated that directional pores can be introduced into water‐based materials via freeze‐drying [40]. When the dopant precursor contacts a host material with directional pores, it preferentially diffuses along the pore channels during sintering, forming a concentration gradient which is crucial to study potential interaction mechanisms at different concentrations.

Unlike conventional silver‐doping approaches that rely on homogeneous mixing or post‐sintering addition, this work proposes and demonstrates an innovative fabrication strategy that integrates dual‐material co‐extrusion with freeze‐drying, enabling the construction of a macroscopically well‐defined and oriented Ag‐YBCO interfacial architecture. On the aqueous YBCO precursor side, the directional growth of ice crystals during freeze‐drying is deliberately exploited to introduce a vertically aligned porous structure perpendicular to the Ag‐YBCO interface. This engineered interfacial architecture markedly alters the transport behavior of silver during subsequent high‐temperature sintering, allowing Ag^+^ ions to undergo spatially confined and directionally controlled rapid diffusion along the aligned pore channels, followed by in situ solid‐solution reactions on both sides of the pore walls. Experimental results demonstrate that Ag^+^ ions diffusing into the Y123 lattice predominantly substitute for Cu(1) sites, leading to the formation of a [001]‐oriented YBCAO solid‐solution phase. First‐principles calculations further reveal that this preferred orientation originates from anisotropic lattice relaxation induced by Ag^+^ substitution: the formation of Ag–O bonds drives an energetically favorable adjustment of the crystal lattice along the *c* axis, rendering sustained growth along the [001] direction crystallographically more stable. The subsequently formed Y123 phase exhibits a high degree of lattice compatibility with the pre‐oriented YBCAO solid‐solution phase, giving rise to coherent interfaces at their boundaries. These interfaces act as effective “templates” for orientation transfer, thereby inducing newly formed Y123 grains to inherit the [001] orientation and enabling the development of overall textured growth. Meanwhile, silver that does not enter the lattice continues to diffuse toward grain boundaries, where it promotes grain bridging and the formation of a continuous intergranular network. The synergistic effects of enhanced crystallographic texture and grain‐boundary bridging not only markedly improve the superconducting properties of the composite but also alter the dominant fracture mode from intergranular to transgranular cracking. Overall, by regulating the coupled relationships among pore architecture, diffusion pathways, and solid‐solution reactions at the macroscopic interfacial scale, this work systematically elucidates a previously unrecognized texture formation mechanism in the Ag‐YBCO system. This mechanism originates from solid‐solution doping, proceeds through orientation transfer mediated by coherent interfaces, and ultimately leads to optimized macroscopic properties. We propose a solid solution‐driven texture formation mechanism, defined as a process in which dopant‐induced solid‐solution phases with a preferred crystallographic alignment act as templates, guiding the subsequent oriented growth of the superconducting phase. Beyond clarifying the physical origin of solid‐solution‐driven texture formation in Ag‐doped YBCO, this work highlights the role of macroscopic interfacial architecture in influencing dopant diffusion and phase evolution, thereby providing insights into texture control strategies in multiphase REBCO superconductors.

## 2 Results

### 2.1 Identification of the YBa_2_Cu_3‐x_Ag_x_O_7‐δ_ Solid Solution Phase

**FIGURE 1:**
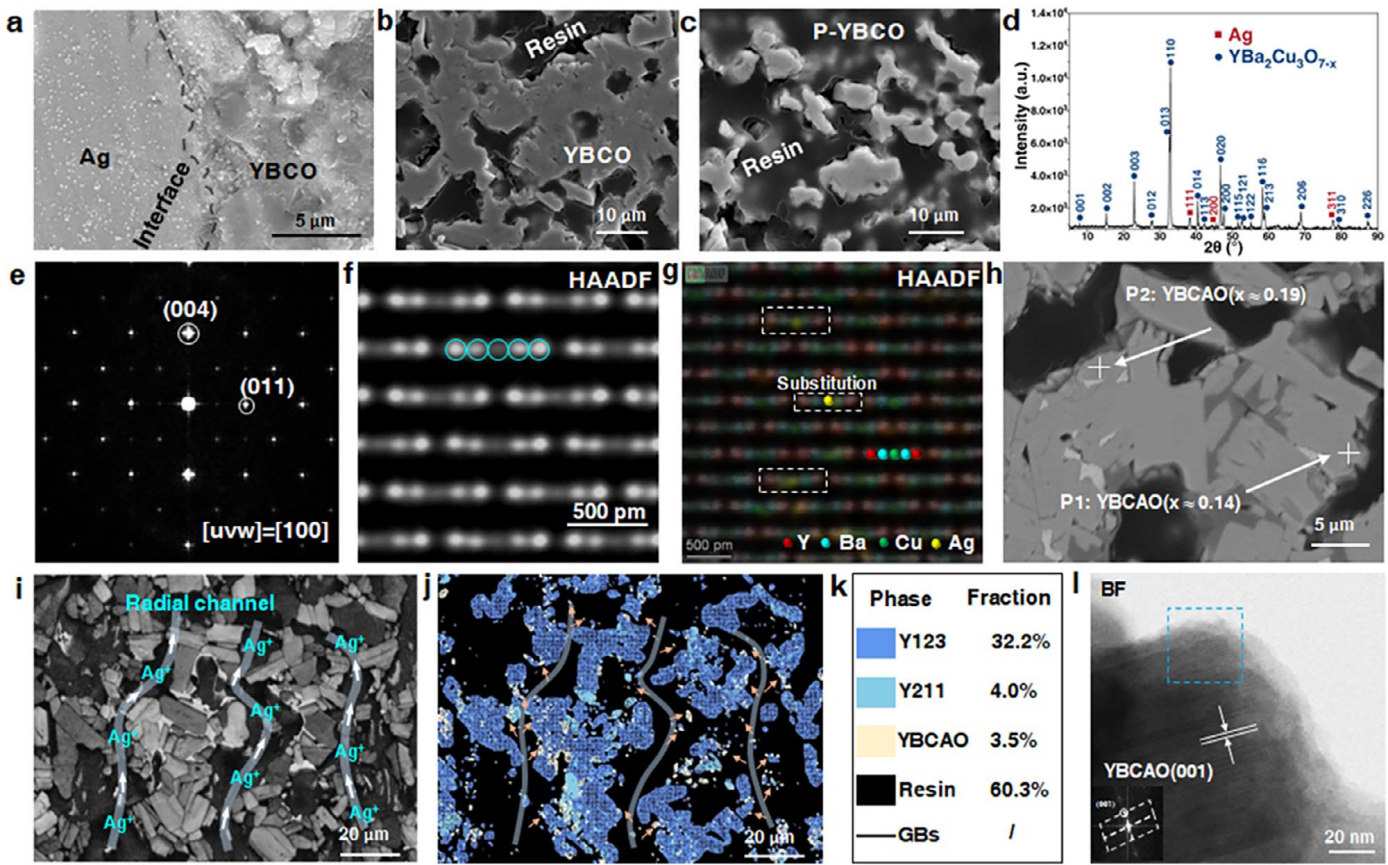
Multiscale structural and compositional identification of the Ag‐induced substitutional solid‐solution YBa_2_Cu_3‐x_Ag_x_O_7‐δ_ (YBCAO) phase in the YBCO‐Ag composite. (a) SE image of the Ag‐YBCO interface showing a tightly bonded interface without pores or voids. (b) SE image of the YBCO region revealing an interconnected network structure formed by bridged YBCO grains. (c) SE image of pure YBCO (P‐YBCO) without Ag composite, displaying dispersed granular particles. (d) XRD patterns and Rietveld refinement results of the composite, indicating Y123 as the dominant crystalline phase with refined lattice parameters of *a* = 3.8202 Å, *b* = 3.8807 Å, and *c* = 11.6798 Å, together with a minor metallic Ag phase, while no distinct diffraction peaks attributable to other Ag‐related secondary phases are detected. (e) Fast Fourier transform (FFT) pattern obtained from the YBCO region, confirming that the electron beam is aligned along the [100] zone axis. (f) STEM‐HAADF image acquired along the [100] zone axis, revealing a characteristic periodic atomic‐plane grouping in which every five atomic planes form a repeating motif. (g) Atomic‐resolution HAADF image of the selected region in (f), in which Ag atoms (highlighted in yellow) are unambiguously identified at Cu atomic columns in multiple unit cells, providing direct evidence of substitutional incorporation of Ag at Cu sites within the Y123 lattice. (h) BSE image for EPMA point analysis, detecting YBCAO distributed at the periphery of YBCO grains. (i) EBSD band contrast map revealing radially aligned pores designed by freeze‐drying. (j) EBSD phase map indicating the predominant distribution of YBCAO along the channel walls, resulting from a solid solution substitution reaction between Ag^+^ and the primary Y123 phase, facilitated by rapid Ag^+^ diffusion along the pores at high temperature. (k) Associated color legend with phase fractions. (l) BF‐STEM image of YBCAO(001) (Figure S3).

To investigate the interactions between Ag and YBCO in depth, an Ag‐YBCO composite structure was fabricated using a dual‐material co‐extrusion combined with a freeze‐drying process. The overall procedure includes precursor powder preparation, slurry formulation, co‐extrusion, freeze‐drying, and co‐sintering (Figure S1). During the low‐temperature cold casting stage, ice crystals grew directionally within the water‐based YBCO precursor. Upon drying, sublimation of the ice crystals resulted in the formation of directional pores within the structure, which serve as diffusion channels for guiding elemental transport. After high‐temperature sintering, the composite structure was infiltrated with epoxy resin to facilitate polishing and surface preparation. Scanning electron microscopy (SEM) characterization confirmed a dense and defect‐free interface between YBCO and Ag, exhibiting no voids, cracks, or pores, and showing a continuous and uniform bonding state (Figure 1a). The YBCO grains in the composite form an interconnected network (Figure 1b), contrasting with the dispersed granular morphology of naturally dried pure YBCO (P‐YBCO; Figure 1c). The latter exhibits lower packing density, attributed to the intrinsic brittleness of YBCO without Ag‐doping reinforcement.

The YBCO‐Ag system may contain five possible phases (Table S1). Rietveld refinement of X‐ray diffraction (XRD) patterns collected from the composites (Figure 1d) indicates that Y123 is the dominant crystalline phase, with refined lattice parameters of *a* = 3.8202 Å, *b* = 3.8807 Å, and *c* = 11.6798 Å. In addition to Y123, only a minor amount of metallic Ag is detected, while no diffraction peaks corresponding to other Ag‐related secondary phases are resolvable. Given that XRD does not unambiguously identify Ag‐doping‐related phases, focused ion beam (FIB) milling was employed to extract lamellae from the YBCO regions for atomic‐scale characterization. Spherical aberration‐corrected high‐angle annular dark‐field scanning transmission electron microscopy (Cs‐corrected HAADF‐STEM) was then used to probe the local atomic structure. When the electron beam is aligned along the [100] zone axis (Figure 1e), the atomic lattice exhibits a characteristic periodic grouping, in which every five atomic planes form a repeating motif (Figure 1f). Atomic‐resolution contrast analysis reveals that this five‐plane sequence corresponds to Y–Ba–Cu–Ba–Y. Notably, Ag atoms (highlighted in yellow) are clearly observed to occupy Cu atomic columns in multiple unit cells within the selected region (Figure 1g), providing direct evidence that Ag substitutes for Cu sites within the Y123 lattice.

To quantitatively determine the local chemical composition, electron probe microanalysis (EPMA) point measurements were performed. At position P1, the measured atomic percentages are Y 7.76, Ba 15.10, Ag 1.07, Cu 22.25, and O 53.82. The combined Ag and Cu content is exactly three times the Y content, consistent with the stoichiometry YBa_2_Cu_3‐x_Ag_x_O_7‐δ_, corresponding to an Ag substitution level of *x* ≈ 0.14 (Figure 1h). Similarly, Ag substitution levels of *x* ≈ 0.19 and *x* ≈ 0.17 are obtained at positions P2 and P3, respectively (Figure 1h; Figure S2a). Importantly, P1‐P3 are all located near the periphery of YBCO grains, whereas no Ag signal is detected in the grain interior regions (P4–P6), where only the Y123 phase is identified (Figure S2b,c and Table S2). Due to the small difference in average atomic number (Δ*Z*) between Y123 and YBCAO, backscattered electron (BSE) imaging does not exhibit discernible compositional contrast between the two phases. In general, BSE contrast becomes detectable only when Δ*Z* exceeds approximately 0.1*Z* [41], where *Z* is the average atomic number of the matrix. For Y123 (*Z* ≈ 22.62) and YBCAO (*Z* ≈ 22.62 + 1.38*x*), this criterion would only be satisfied for *x* > 1.64, well beyond the substitution range investigated here.

Despite the clear atomic‐ and compositional‐level evidence for Ag substitution, no distinct diffraction peaks attributable to a YBCAO phase are resolved in the laboratory XRD patterns. This absence can be rationalized by the following factors. First, conventional laboratory XRD probes the long‐range average crystal structure and exhibits limited sensitivity to low‐concentration substitutional solid solutions. Second, at the relatively low substitution levels identified by EPMA (*x* ≈ 0.14–0.19), the lattice parameters of the YBCAO solid solution remain very close to those of the parent Y123 phase, resulting in substantial overlap of the corresponding Bragg reflections. Third, local lattice distortions induced by Ag‐for‐Cu substitution introduce microstrain and peak broadening, further reducing the distinguishability of the solid‐solution phase.

Electron backscatter diffraction (EBSD) was further employed to analyze the spatial distribution of phases within the YBCO regions. EBSD band contrast maps clearly reveal the aligned pore‐channel architecture formed during freeze‐drying (Figure 1i), while the YBCAO phase is preferentially distributed along the walls of these radially aligned channels (Figure 1j). This spatial correlation indicates that Ag^+^ ions diffuse efficiently along the oriented pore channels at high temperature and undergo atomic exchange reactions with pre‐formed Y123 near the pore surfaces. During this process, Ag^+^ incorporates into the Y123 lattice by substituting for Cu^2+^ at Cu(1) sites, giving rise to a substitutional solid solution identified as the YBCAO phase [20, 21, 28, 35]. Phase fraction analysis within the selected area indicates that YBCAO accounts for approximately 3.5%, while the Y211 phase occupies about 4% (Figure 1k).

Finally, nanoscale structural characterization of the YBCAO phase was performed using bright‐field scanning transmission electron microscopy (BF‐STEM) imaging. Well‐defined lattice fringes corresponding to the (001) planes of YBCAO are clearly observed (Figure 1l). Fast Fourier transform (FFT) and inverse FFT (IFFT) analyses of the selected region (Figure S3a,b) yield an interplanar spacing of 11.71 Å for the (001) planes (Figure S3c), which is slightly larger than that of the Y123 phase. This modest lattice expansion along the *c*‐axis can be attributed to the larger ionic radius of Ag^+^ relative to Cu^2+^, leading to local lattice relaxation [20, 28, 35]. Further STEM‐EDS mapping confirms a uniform distribution of Y, Ba, Cu, Ag, and O within the YBCAO regions (Figure S3d,e). Collectively, these observations indicate that Ag is incorporated into the Y123 lattice via atomic‐scale substitution while remaining compositionally homogeneous at the nanoscale, thereby forming a chemically uniform substitutional solid‐solution phase.

This finding provides important clarification in the context of previous experimental reports, where the feasibility of Ag^+^ lattice substitution has remained controversial. Some researchers, based on experimental observations, have argued that Ag^+^ ions are unlikely to achieve effective lattice substitution [14, 15, 16]. In fact, the formation of the YBCAO phase is strictly dependent on specific reactant systems and heat treatment conditions. The process requires yttrium, barium, and copper sources along with Ag powder (or silver substrates) as starting materials, and must be carried out at heat treatment temperatures below the peritectic reaction temperature of Y123, typically in the range of 900°C–920°C. This temperature range is chosen because the introduction of Ag significantly lowers the peritectic reaction temperature of Y123 [34]. This enables Ag atoms to migrate effectively into Y123 unit cells during phase formation, ultimately forming the substitutional solid solution YBCAO. Furthermore, the limited resolution of microscopic techniques at the time may have affected accurate determination of Ag's doping state. Ch. Zhang et al. [20] reported that the saturation level of Ag in the YBCAO phase (*x*_max_) was only 0.023. Their study directly mixed Y123 superconducting powder with Ag, bypassing the Y123 phase formation process, which hindered the effective incorporation of Ag into the lattice. Additionally, the sintering temperature they employed (950°C) exceeded the optimal range for YBCAO phase formation, further limiting Ag solubility. Subsequent studies have demonstrated higher Ag solubility [21, 35], showing that optimizing sintering conditions (e.g., reducing sintering temperature or adjusting oxygen partial pressure) can effectively enhance the incorporation of Ag into the YBCO lattice.

### 2.2 First‐Principles Calculation of the Structural Optimization for YBa_2_Cu_3‐x_Ag_x_O_7‐δ_

Ag^+^ preferentially substitutes at the Cu(1) sites in the Y123 lattice, primarily due to the higher compatibility of Cu(1) sites in terms of both structure and charge. Structurally, compared with the relatively rigid Cu(2) sites within the CuO_2_ planes, the chain‐like Cu(1) sites exhibit greater structural tolerance. Their local coordination environment can adaptively distort to accommodate the larger ionic radius of Ag^+^, thereby alleviating the stress induced by lattice mismatch. From the perspective of charge balance, Cu(2) typically maintains a stable +2 oxidation state, whereas Cu(1) can tolerate variable oxidation states ranging from +1 to +3. When +1 Ag^+^ substitutes for +2 Cu(1), the resulting local charge imbalance can be effectively compensated through the formation of nearby oxygen vacancies. This unique charge self‐compensation mechanism further stabilizes the occupation of Ag^+^ at the Cu(1) sites [12, 20, 21, 32, 35].

Based on the substitution mechanism described above, five YBa_2_Cu_3_O_7−δ_ supercell models were constructed for computational analysis to systematically characterize structural features under different oxygen contents and vacancy configurations [42, 43, 44, 45]. Three representative idealized ordered structures were first considered, including 2 × 3 × 1 YBa_2_Cu_3_O_7_ (Figure S4a), 3 × 1 × 1 YBa_2_Cu_3_O_6.5_ (Figure S4b), and 2 × 3 × 1 YBa_2_Cu_3_O_6_ (Figure S4c), to capture the evolution of the Cu–O chain framework from fully oxygenated to strongly oxygen‐deficient states. In orthorhombic YBa_2_Cu_3_O_7_ and YBa_2_Cu_3_O_6.5_, the crystal structure exhibits a characteristic layered stacking along the *c* axis, consisting of alternating Cu(1)–O(2) chains, Ba–O(1) layers, CuO_2_ (Cu(2)–O(3)–O(4)) planes, and Y layers. By contrast, YBa_2_Cu_3_O_6_, an antiferromagnetic Mott insulator, lacks continuous Cu–O chains [42]. Considering that oxygen vacancies in practical samples are generally distributed in a partially disordered manner rather than forming perfectly periodic arrangements, two additional non‐integer oxygen‐content models were introduced to approximate experimentally relevant vacancy statistics: 2 × 2 × 1 YBa_2_Cu_3_O_6.8_ (Figure S4d), dominated by chain‐end vacancies, and 2 × 2 × 1 YBa_2_Cu_3_O_6.75_ (Figure S4e), containing both chain‐end and in‐chain vacancies. These models preserve oxygen contents within the experimental range while introducing distinct local vacancy environments, thereby enabling a more realistic description of oxygen‐vacancy distributions in real YBa_2_Cu_3_O_7−δ_ systems.

To systematically investigate the microscopic effects of the coupled evolution between Ag doping level and oxygen‐vacancy concentration on the structural stability and lattice relaxation behavior of YBa_2_Cu_3_O_7−δ_, one Ag atom was introduced into each of the YBa_2_Cu_3_O_7_, YBa_2_Cu_3_O_6.5_, and YBa_2_Cu_3_O_6_ supercell configurations. In addition, three Ag atoms were introduced to substitute Cu(1) sites in the YBa_2_Cu_3_O_6.8_ and YBa_2_Cu_3_O_6.75_ supercells. This modeling strategy resulted in five Ag‐doped structures: YBa_2_Cu_2.8333_Ag_0.1667_O_7‐δ_ (δ = 0, 0.5, and 1), YBa_2_Cu_2.85_Ag_0.15_O_6.8_, and YBa_2_Cu_2.8125_Ag_0.1875_O_6.75_. In these models, the effective Ag doping level *x* spans the range of 0.15–0.19, which is in excellent agreement with the Ag solid‐solution range determined experimentally by EPMA (*x* ≈ 0.14–0.19). This compositional consistency ensures a close correspondence between the computational models and the real material system.

**FIGURE 2:**
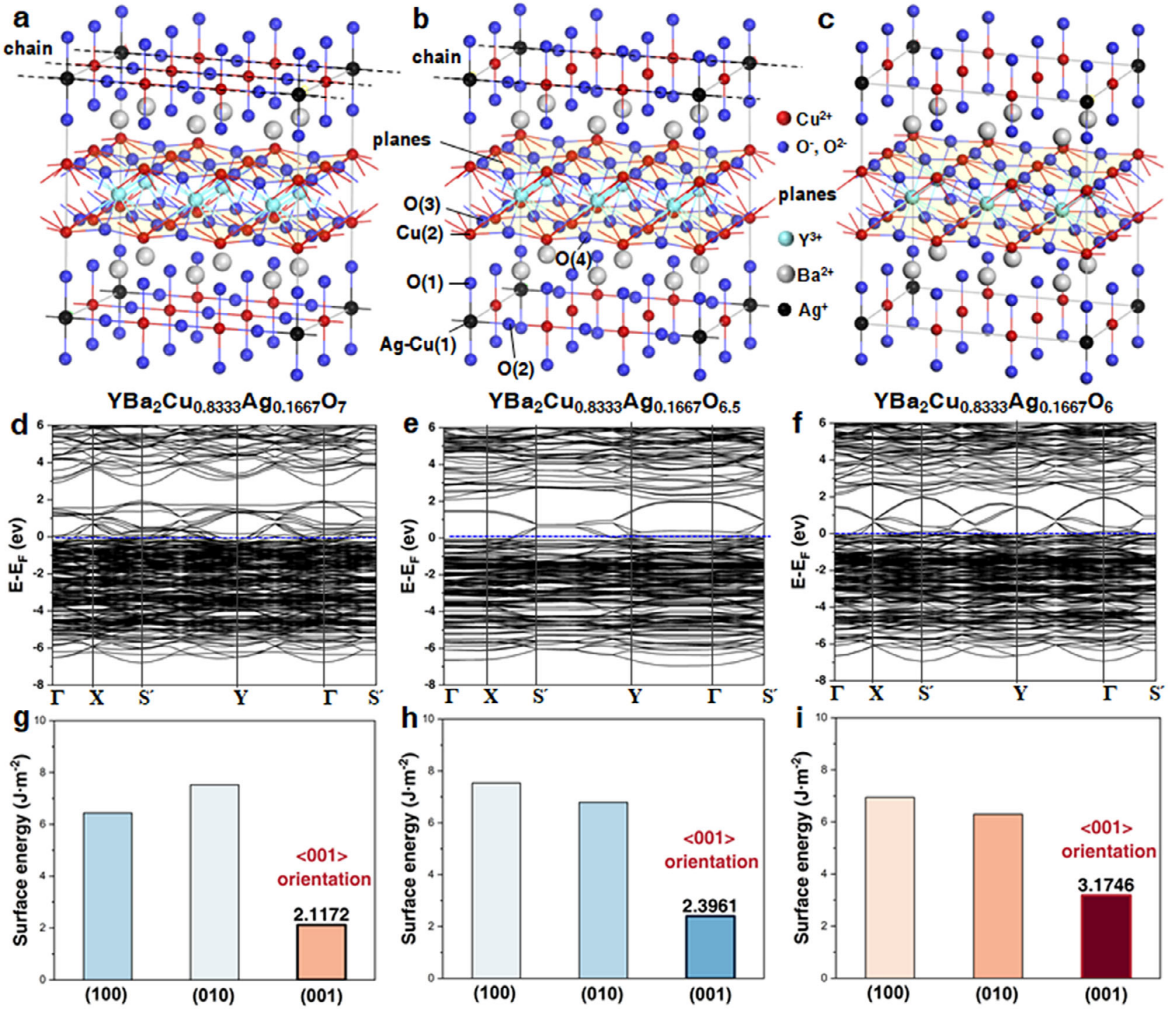
First‐principles structural optimization, electronic structure, and surface energy analysis of Ag‐doped YBa_2_Cu_3_O_7−δ_ systems. (Section S1). (a–c) Optimized crystal structures of YBa_2_Ag_0.1667_Cu_2.8333_O_7‐δ_ with δ = 0, 0.5, and 1, respectively, illustrating Ag substitution at the Cu(1) site and the associated local lattice relaxation. With increasing oxygen deficiency, pronounced local distortions develop in the Cu–O chain region, accompanied by enhanced lattice relaxation along the *c* axis (Tables S3 and S4). (d–f) Corresponding electronic band structures of YBa_2_Ag_0.1667_Cu_2.8333_O_7‐δ_ for δ = 0, 0.5, and 1. The band gap near the Fermi level progressively narrows with increasing δ and nearly closes at δ = 1, indicating a transition from semiconducting to quasi‐metallic electronic characteristics driven by the cooperative effects of Ag doping and oxygen‐vacancy–induced Cu–O chain disruption. (g–i) Calculated surface energies of the (100), (010), and (001) low‐index crystallographic planes for YBa_2_Ag_0.1667_Cu_2.8333_O_7‐δ_ with δ = 0, 0.5, and 1, respectively. In all cases, the (001) surface exhibits the lowest surface energy, suggesting its thermodynamic preference and a tendency for preferential crystal growth along the ⟨001⟩ direction.

We employed the CASTEP module [46] in Materials Studio [47] to perform first‐principles‐based structural optimization and energy calculations for the five Ag‐doped Y123 supercell models (Section S1, Figure 2a–c; Figure S5a,b). Under zero external pressure, the equilibrium configurations and the corresponding lattice parameters before and after optimization are summarized in Table S3. For the YBa_2_Ag_0.1667_Cu_2.8333_O_7‐δ_ (δ = 0, 0.5, and 1) supercell models, the Ag atom occupies symmetry‐equivalent Cu(1) sites located at the eight corners of the supercell. Under periodic boundary conditions, this configuration is equivalent to substitution at a single Cu(1) site, corresponding to an effective occupation fraction of *x* = 0.1667. Structural optimization reveals that Ag incorporation induces pronounced local lattice distortions across all oxygen contents, predominantly manifested as lattice relaxation along the *c* axis (Table S4). In the fully oxygenated structure (δ = 0), Ag doping leads to local rearrangement in the Cu–O chain region. Specifically, the O(1) atom is displaced by approximately 0.13 Å, accompanied by a deviation of 0.93° in the Ag–O(1) bond angle, while the O(2) atom exhibits a displacement of 0.08 Å with a corresponding Ag–O(2) bond‐angle deviation of 0.52°. These local structural adjustments collectively result in an expansion of the *c*‐axis lattice parameter by 0.56% relative to the undoped system. When the oxygen deficiency increases to δ = 0.5, Ag atoms still preferentially occupy Cu(1) sites coordinated with O(2), likely due to favorable charge matching and geometric compatibility of the local coordination environment. The introduction of oxygen vacancies reduces the structural rigidity of the Cu(1)–O(2) chains, facilitating accommodation of the larger‐radius Ag^+^ ions. In this configuration, the displacements of O(1) and O(2) increase to 0.14 and 0.09 Å, respectively, leading to a further enhancement of the *c*‐axis expansion to 0.93%. This behavior indicates a pronounced amplification of Ag‐induced lattice relaxation under oxygen‐deficient conditions. In the highly oxygen‐deficient system (δ = 1), the nature of structural relaxation changes markedly. The displacement of the O(1) atom increases to 0.21 Å, and the Ag–O(1) bond‐angle deviation reaches 1.54°, indicating that bond‐angle distortion becomes the dominant mode of structural adjustment. Correspondingly, the relative expansion of the *c*‐axis lattice parameter further increases to 1.07%. This behavior primarily originates from the complete disruption of the Cu–O chains under highly oxygen‐deficient conditions, which significantly reduces the coordination number of the Cu(1) site and transforms the local coordination polyhedron from a chain‐like geometry to a simpler coordination motif. The resulting weakening of bonding strength along the *c* axis renders the lattice more susceptible to relaxation and expansion upon Ag^+^ incorporation.

Variations in Ag content also exert a significant influence on local distortion and lattice response. In YBa_2_Cu_2.85_Ag_0.15_O_6.8_ with a lower Ag concentration, the displacements of O(1) and O(2) reach 0.34 and 0.24 Å, respectively, accompanied by the most pronounced *c*‐axis–oriented relaxation (1.32%). By contrast, when the Ag content increases to YBa_2_Cu_2.8125_Ag_0.1875_O_6.75_, the corresponding atomic displacements and bond‐angle deviations are partially suppressed, and the *c*‐axis expansion decreases to 0.65%. These results indicate that the lattice response to Ag doping is governed by the cooperative interplay between oxygen‐vacancy concentration and the local coordination environment.

The calculated band structures further reveal the cooperative modulation of the electronic structure by Ag doping and oxygen vacancies (Figure 2d–f). For δ = 0, a pronounced band gap is observed near the Fermi level, with a gap width of *E*_g_ = 0.824 eV, indicating that Ag substitution at the Cu(1) site does not introduce significant impurity states. The electronic structure thus remains semiconducting in nature, dominated by contributions from the Cu–O chains and CuO_2_ planes. As the oxygen deficiency increases to δ = 0.5, the bands near the Fermi level become markedly compressed, and the band gap decreases sharply to 0.084 eV. This evolution reflects oxygen‐vacancy–induced electronic redistribution and the progressive disruption of Cu–O chain continuity, driving the electronic states toward a quasi‐metallic character. In the highly oxygen‐deficient system (δ = 1), the band gap further converges to 0.079 eV and is nearly closed, indicating the formation of high‐density electronic states in the vicinity of the Fermi level. This behavior is closely associated with the complete destruction of the Cu–O chains and the reconstruction of the local coordination environment at the Cu(1) sites, which significantly enhances the degree of electronic‐state delocalization.

We further constructed surface models corresponding to the (001), (010), and (100) low‐index crystal planes and systematically calculated the surface energies of each facet. For each of the five Ag‐doped structures, the bulk unit‐cell energy (*E*_bulk_) and the energies of the corresponding surface slab models (*E*_slab_) for the three crystallographic planes were calculated with the numerical results summarized in Table S5. The surface energy (γ) was evaluated using the Equation (1) [48]. Where *n*_slab_ denotes the number of bulk unit cells contained in the surface slab model, and *A* represents the constructed surface area. For the (001) plane, *A* is given by the product of the lattice constants *a × b*. Analysis of the calculated surface energies for different planes in the three structures reveals that the (001) plane consistently exhibits the lowest surface energy (Figure 2g–i; Figure S5c,d), indicating its superior crystallographic stability. Therefore, during material growth, the crystal tends to stack sequentially along the (001) planes, resulting in a preferred growth orientation along the ⟨001⟩ direction (Figure S6 and S7).

### 2.3 Microstructural Characterization of Y123/ YBa_2_Cu_3‐x_Ag_x_O_7‐δ_ Coherent Interfaces

**FIGURE 3:**
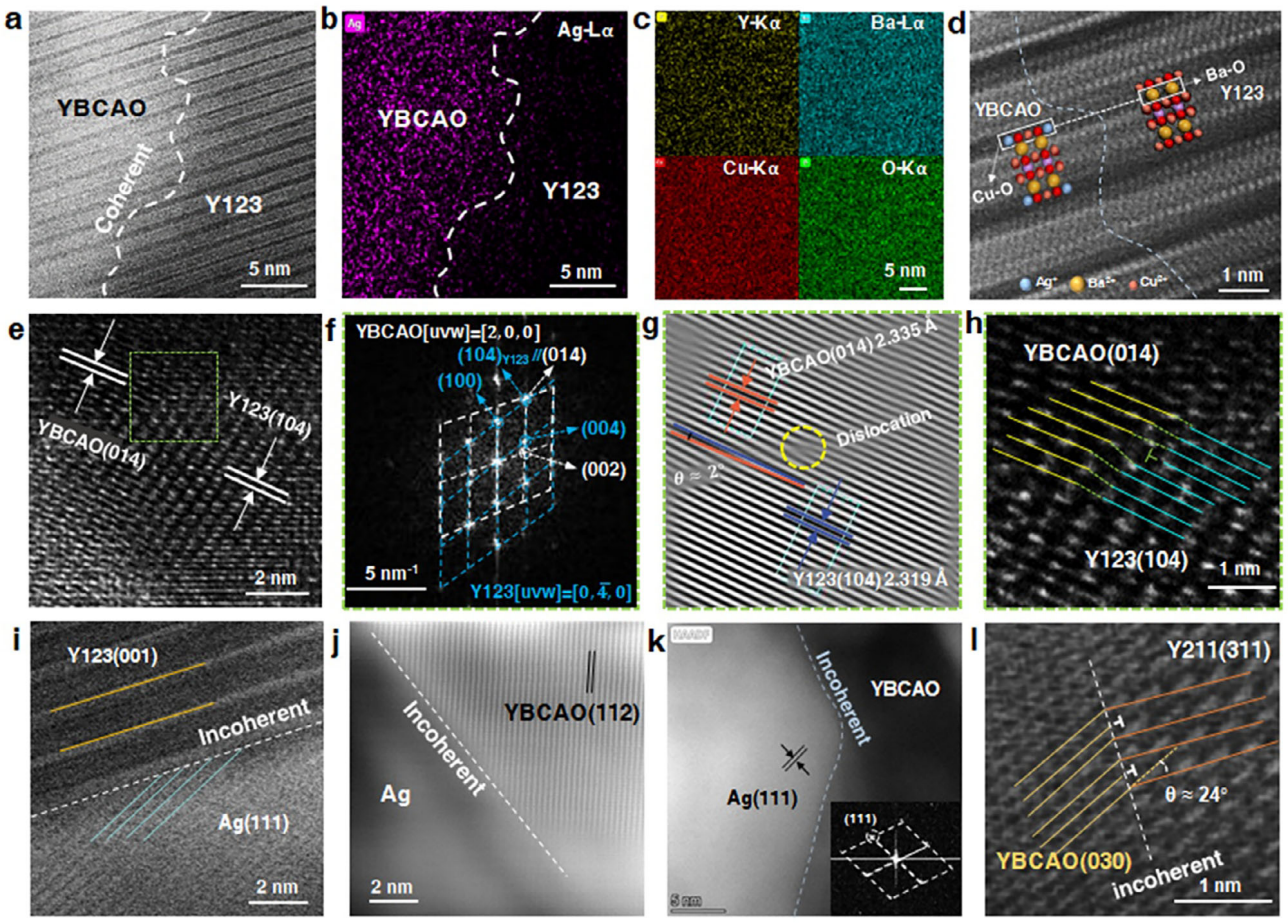
Microstructural characterization of the Y123/YBCAO coherent interface and the incoherent interfaces of Y123/Ag, YBCAO/Ag, and YBCAO/Y211. (a) HAADF‐STEM image. (b,c) Nanoscale STEM‐EDS mapping showing a localized distribution of Ag, which is highly enriched on one side of the interface, whereas Y, Ba, Cu, and O are uniformly distributed throughout the image. This confirms the phase distribution and interface location of YBCAO and Y123, verifying the coherence between YBCAO and Y123. (d) HAADF image of the YBCAO/Y123 interface, showing a continuous atomic transition across the interface, with the Ba‐O layers of Y123 perfectly aligned with the Cu‐O chains of YBCAO.  (e) HRTEM image of the interfacial region. (f) Corresponding FFT image. (g) IFFT image. (h) Atomic arrangement at the YBCAO(014)/Y123(104) coherent interface (Figure S8). (i) Incoherent interface of Y123(001)/Ag(111), with an interfacial misorientation of approximately 25°. (j) HAADF image of incoherent interface between YBCAO(112) and Ag. (k) HAADF image of incoherent interface between YBCAO and Ag(111). (l) HRTEM image of incoherent interface between YBCAO(030) and Y211(311) (Figure S9).

We employed high‐angle annular dark‐field scanning transmission electron microscopy (HAADF‐STEM) to characterize the interfacial relationship between the YBCAO phase and other phases in the system at the atomic scale. A coherent phase boundary was observed between the YBCAO and Y123 phases (Figure 3a). Nanoscale STEM‐EDS mapping revealed that Ag exhibited a localized distribution, being highly enriched on one side of the interface, whereas Y, Ba, Cu, and O were uniformly distributed throughout the entire region (Figure 3b,c), thereby confirming the phase distribution and interfacial location of YBCAO and Y123. Figure 3d reveals the atomic‐scale features at the interface between the YBCAO and Y123 phases. In HAADF images, atomic sites with higher atomic numbers appear brighter and more distinct. Consequently, the brightest upper and lower atomic rows in the Y123 region correspond to the Ba‐O layers. In the YBCAO region, the substitution of Cu^2+^ by Ag^+^ enhances the atomic‐site brightness of the Cu‐O chain layer. Atomic‐resolution analysis confirms a continuous transition at the YBCAO/Y123 interface, where the Ba‐O layers of Y123 maintain perfect alignment with the Cu‐O chains of YBCAO without dislocations or atomic displacements, providing definitive evidence of their coherent relationship (Figure 3d). FFT processing of the high‐resolution TEM (HRTEM) image (Figure 3e) generated two distinct diffraction patterns (Figure 3f). Detailed IFFT processing and IFFT live profiles (Figure S8) confirmed lattice spacings of 2.319 Å for Y123(104) and 2.335 Å for YBCAO(014), demonstrating their near‐parallel alignment (*θ* ≈ 2°) (Figure 3g). A misfit dislocation is observed at the interfacial region between lattice fringes, resulting from localized lattice distortions induced by Ag^+^ substitution. Figure 3h reveals two bright spot arrays and one vacancy, demonstrating atomic ordering at the coherent Y123(104)/YBCAO(014) interface. The lattice misfit (*δ*) can be evaluated by the Equation (2) [49]. With *δ* as low as 0.68%, the lattices of the two phases are nearly perfectly matched, resulting in low interfacial energy and high atomic arrangement continuity. The lattices of the two phases transition continuously, forming a coherent interface.

We investigated the interfacial relationships among YBCAO/Ag, YBCAO/211, and Y123/Ag. HAADF‐STEM analysis indicates that the (111) planes of Ag terminate at the (001) planes of Y123 (Figure 3i). This orientation differs from the parallel relationship between Y123(001) and Ag(111) reported by M. E. Tidjani et al. [17], who deposited Ag on YBCO thin films via vacuum evaporation. In our study, a misorientation of approximately 25° exists between Ag(111) and Y123(001). This discrepancy is likely related to the interface preparation method, as the YBCO thin films used by them exhibited strong crystallographic orientation, whereas the composite samples in our study formed under more complex interfacial conditions. Furthermore, a clear incoherent relationship is observed at the YBCAO/Ag interface (Figure 3j). When the sample is tilted to align the electron beam along the [110] zone axis of Ag, the (111) planes of Ag are clearly visible, whereas the YBCAO phase appears blurred due to the orientation mismatch, further confirming the incoherent nature of the interface (Figure 3k). Similarly, the YBCAO/Y211 interface is also incoherent (Section S2 and Figure S9), with a lattice mismatch of 51.34% between YBCAO(030) and Y211(311) (Figure 3l). These interfacial structural features indicate that, within the Ag‐YBCO composite system, only the YBCAO/Y123 interface exhibits a coherent relationship. This coherence ensures crystallographic continuity and an ordered transition across the interface, thereby enhancing interphase bonding stability and potentially transmitting orientation information during phase transformation or growth to influence the system's texture evolution and microstructure.

### 2.4 Crystallographic Orientation Consistency and Solution‐Driven Texture Mechanism

**FIGURE 4:**
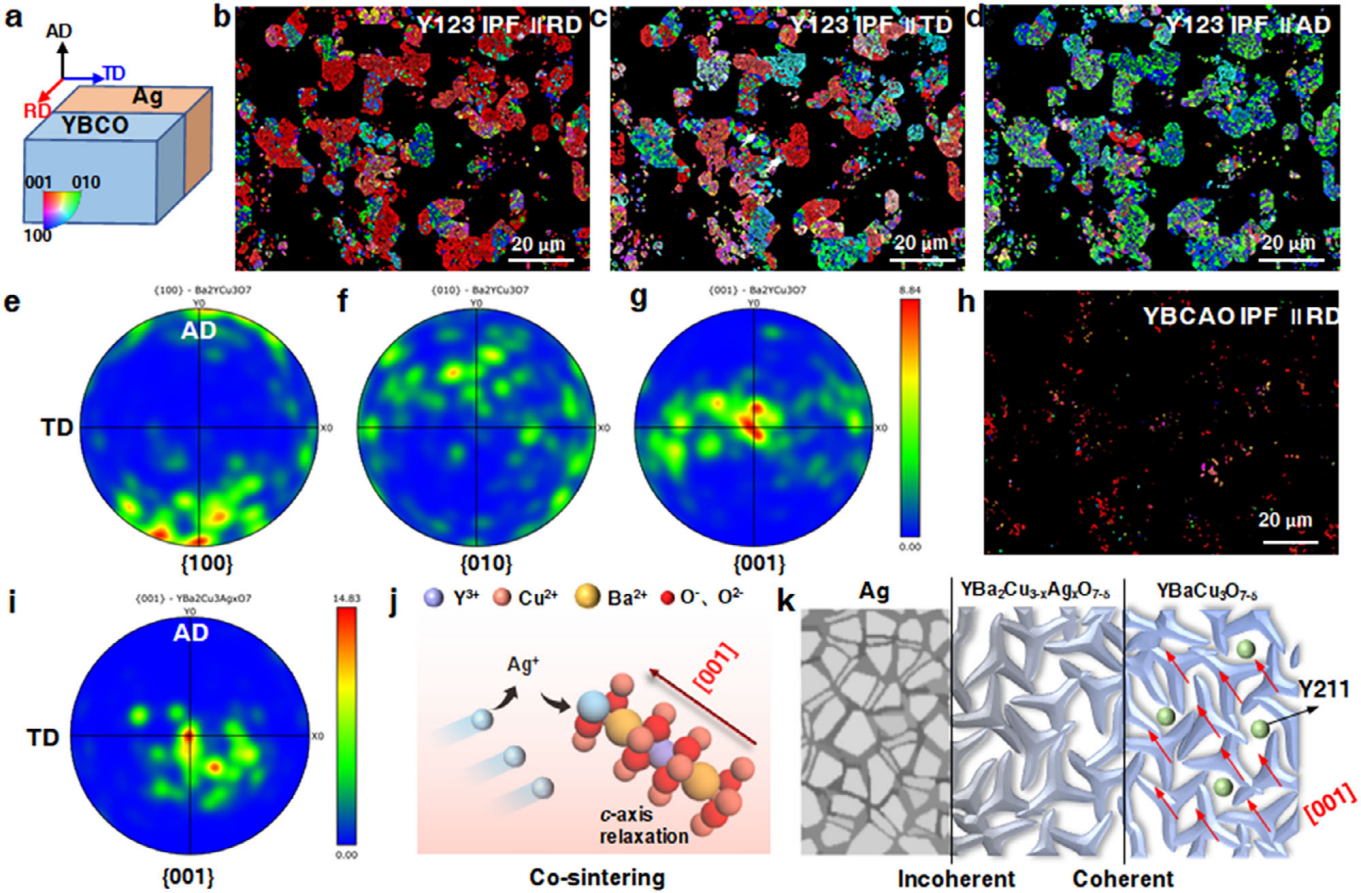
Grain orientation analysis of the Ag‐YBCO composite. (a) Sample coordinate system, where RD is perpendicular to the Ag‐YBCO interface, AD is perpendicular to the sample cross‐section, and TD is perpendicular to RD. The inset at the lower‐left corner shows the corresponding inverse pole figure (IPF) color legend. (b) IPF map of the Y123 phase along RD, showing the [001] orientation in red. (c) IPF map of the Y123 phase along TD. (d) IPF map of the Y123 phase along AD, showing the [100] orientation in blue and [010] orientation in green. (e) Pole figure (PF) of the {100} planes of Y123, with poles concentrated near the AD direction. (f) PF of the {010} planes of Y123. (g) PF of the {001} planes of Y123, with poles aligned along RD, indicating that the {001} plane normals are parallel to RD. (h) IPF map of the YBCAO phase along RD, showing the [001] orientation in red. (i) PF of the {001} planes of YBCAO, with poles along RD, indicating high consistency with the Y123 grain orientation. (j) Solid‐solution process of Ag^+^ substituting Cu(1) sites during high‐temperature co‐sintering; the solid‐solution drives preferential growth of YBCAO along the [001] orientation. (k) Schematic of interface relationships: Ag/YBCAO forms an incoherent interface, whereas YBCAO/Y123 forms a coherent interface, transmitting orientation information so that subsequently formed Y123 inherits the [001] orientation. Y211, as a secondary phase, provides flux pinning without exhibiting a preferential orientation.

To systematically investigate the influence of the coherent interface between the YBCAO and Y123 phases on the orientation and microstructure of YBCO, we performed EBSD characterization on the YBCO regions near the Ag‐YBCO interface to evaluate grain orientation behavior and texture features. The sample coordinate system is illustrated in Figure 4a, where the radial direction (RD) is defined perpendicular to the Ag‐YBCO interface, the axial direction (AD) is perpendicular to the sample cross‐section, and the transverse direction (TD) is perpendicular to RD. The inset at the lower‐left corner shows the corresponding inverse pole figure (IPF) color legend, in which each color represents a specific crystallographic orientation.

In the IPF map of the Y123 phase along the RD direction (Figure 4b), the red regions representing the [001] orientation (*c*‐axis) are dominant and widely distributed, indicating a pronounced *c*‐axis preferential orientation along RD. In contrast, the IPF map along the TD direction (Figure 4c) exhibits no significant color clustering, with orientations distributed more randomly, consistent with its tangential geometry perpendicular to RD. The IPF map along the AD direction (Figure 4d) is dominated by blue and green regions representing the [100] and [010] orientations (*a*‐ and *b*‐axes), indicating that Y123 crystals are primarily aligned within the *a‐b* planes along AD. Pole figure (PF) analysis of the Y123 phase further corroborates these orientation relationships (Figure 4e–g). The poles of the {100} planes are concentrated near the AD direction, whereas the poles of the {001} planes are aligned along RD, further confirming that the {001} planes are oriented with their normals parallel to RD. This implies that the *a‐b* planes of Y123 grains tend to tilt along the direction parallel to the YBCO‐Ag interface, leading to a pronounced texture near the interface. Analysis of the grain orientation of the YBCAO phase alone reveals that its IPF map (Figure 4h) and PF (Figure 4i) exhibit crystallographic orientations highly consistent with those of Y123. In contrast, Y211 as a secondary phase, only displays conventional flux pinning behavior and does not exhibit pronounced grain orientation characteristics (Figure S10). For comparison, EBSD characterization was also performed on P‐YBCO samples without Ag. In the same sample coordinate system, the tri‐axial IPF maps of the Y123 phase show a disordered orientation distribution (Figure S11d–f), and the {100}, {010}, and {001} poles in the PF are randomly distributed (Figure S11g–i), indicating no obvious preferential orientation. This disordered crystallographic feature contrasts sharply with the strong texture observed in samples influenced by Ag‐doping.

To assess the effect of deviations from the optimal sintering window on solid‐solution formation and texture quality, EBSD analyses were performed on samples sintered at 900°C under a controlled oxygen atmosphere (O_2_ 100 sccm). Compared with the optimal condition (920°C in air), the Y123 phase remains dominant, but its fraction increases from 32.2% to 41.9%, whereas the YBCAO solid‐solution fraction decreases from 3.5% to 2.7% (Figure S12a–c), indicating reduced Ag incorporation into the Y123 lattice under off‐optimal conditions. Although [001]‐oriented Y123 grains are still present along the RD direction (Figure S12d), their orientation concentration is noticeably weakened. In the AD direction, the dominant [100]/[010] orientations become more dispersed (Figure S12f). Consistently, PFs show that while {001} poles remain roughly aligned with RD, the {100} and {010} poles exhibit significantly reduced alignment along AD compared with the optimal condition (Figure S12g–i), reflecting degraded texture quality.

In addition, the influence of pore architecture on Ag^+^ diffusion efficiency and texture formation was investigated. Distinct pore structures were obtained by adjusting the freeze‐drying conditions (Table S6), and EBSD was employed to characterize pore morphology and crystallographic orientation, thereby elucidating the role of pore architecture in regulating Ag^+^ transport. Under the slow‐freezing condition (−2°C·min^−1^ to −40°C, 2 h), ice crystals grow and interconnect sufficiently, leading to the formation of large, directionally aligned, through‐thickness pore channels after freeze‐drying. As shown in the band contrast images (Figure S13a–c), only three pronounced pore channels are observed within the selected area, each with a characteristic diameter exceeding 20 µm. Although such large interconnected pores facilitate rapid local migration of Ag^+^, the diffusion process becomes excessively fast, preventing Ag^+^ from effectively participating in the solid‐solution reaction at Cu(1) sites. Consequently, the formation efficiency of the YBCAO solid‐solution phase is significantly suppressed. Within the selected region, the area fraction of the YBCAO phase is only 2.1% and is primarily distributed along both sides of the large pore channels. Moreover, the enlarged pore‐dominated regions reduce the effective area available for phase identification to 22.5%. In contrast, fast freezing by liquid‐nitrogen quenching followed by holding at −80°C for 0.5 h produces small, nearly equiaxed pores with diameters below 5 µm but poor interconnectivity (Figure S13d–f). The restricted pore network suppresses long‐range Ag^+^ diffusion, leading to insufficient local supply and similarly limiting solid‐solution formation, with a YBCAO phase fraction of 2.5%. IPF and PF analyses of Y123 grains indicate that neither processing route yields a well‐developed texture (Figures S14 and S15). The slow‐freezing sample exhibits highly dispersed grain orientations, while the fast‐freezing sample shows only weak local alignment, with the {001} pole preferentially oriented along the RD direction but without pronounced texture enhancement.

Overall, both excessively large interconnected pores and overly fine, poorly connected pores are unfavorable for the coupled evolution of Ag^+^ diffusion, solid‐solution reactions, and texture development. In comparison, the intermediate freezing strategy employed in this work (−5°C·min^−1^ to −50°C, 1 h) provides a more balanced pore architecture, offering suitable kinetic conditions for sustained Ag^+^ diffusion and effective solid‐solution formation, thereby facilitating subsequent texture evolution.

Building on this optimized pore design, the YBCO side in our macroscopic Ag‐YBCO composite system was pre‐structured with a directionally aligned porous architecture to systematically investigate these interaction effects. During high‐temperature sintering, three major phase‐forming reactions occur within the system: the formation of the Y123 superconducting phase, the precipitation of the Y211 secondary phase, and the solid‐solution substitution of Cu^2+^ in the nascent Y123 lattice by Ag^+^, leading to the formation of the YBCAO solid solution (Figure 4j). Among these, the solid‐solution process of Ag^+^ plays a critical role in the evolution of the microstructure. Driven by solid solution, the YBCAO phase exhibits pronounced preferential orientation along the [001] direction from the early nucleation stage (Figure 4k). This orientation arises from lattice relaxation induced by Ag^+^ substitution at the Cu(1) sites: the formation of Ag‐O bonds promotes directional adjustment of the lattice along the *c*‐axis, energetically favoring continued growth along [001]. Subsequently formed Y123 grains, having high lattice compatibility with the pre‐oriented YBCAO phase, form coherent interfaces with low interfacial energy. These interfaces further guide the orientated growth of newly formed Y123 grains, allowing them to inherit and extend the [001] orientation of the YBCAO phase. Notably, this Ag‐induced solid‐solution formation and orientation‐transfer process is highly sensitive to sintering temperature and oxygen partial pressure. Deviations from the optimal processing window suppress Ag diffusion and solid‐solution reactions, reduce the formation efficiency of the YBCAO phase, and weaken its templating effect on Y123 grain growth, ultimately leading to a pronounced degradation of the overall texture strength. Regarding the effective Ag doping threshold, our focus is on elucidating the mechanistic role of Ag‐induced solid‐solution formation in driving texture development. EPMA measurements indicate that, under the optimal processing window, the effective Ag substitution level in the Y123 lattice lies within *x* ≈ 0.14‐0.19. Within this range, a YBCAO solid‐solution phase with a preferred [001] orientation is reproducibly formed and acts as an orientation template for subsequent Y123 grain growth. Therefore, the “threshold” identified in this work should be understood as a mechanistic threshold for solid‐solution‐driven texture formation, rather than a universal critical composition value.

The kernel average misorientation (KAM) map of Y123 in the Ag‐YBCO system (Figure S16) shows that 99.4% of the grains have KAM values below 2°, indicating minimal internal strain accumulation during sample construction. The average grain size of Y123 is 1.12 µm (Figure S17a), which is smaller than that of P‐YBCO (1.73 µm; Figure S17b), suggesting that the incorporation of Ag also contributes to grain refinement.

### 2.5 Superconducting Performance and Mechanical Behavior of Ag‐YBCO Composites

For magnetization measurements and micro‐mechanical property testing, cylindrical Ag‐YBCO short samples with diameters of 3∼5 mm were fabricated using a co‐extrusion process with dual‐material nozzles (Figure S18a). After freeze‐drying, radially distributed pores were observed (Figure S18b), indicating that the directional pores formed by this process are all oriented perpendicular to the macroscopic Ag‐YBCO interface.

**FIGURE 5:**
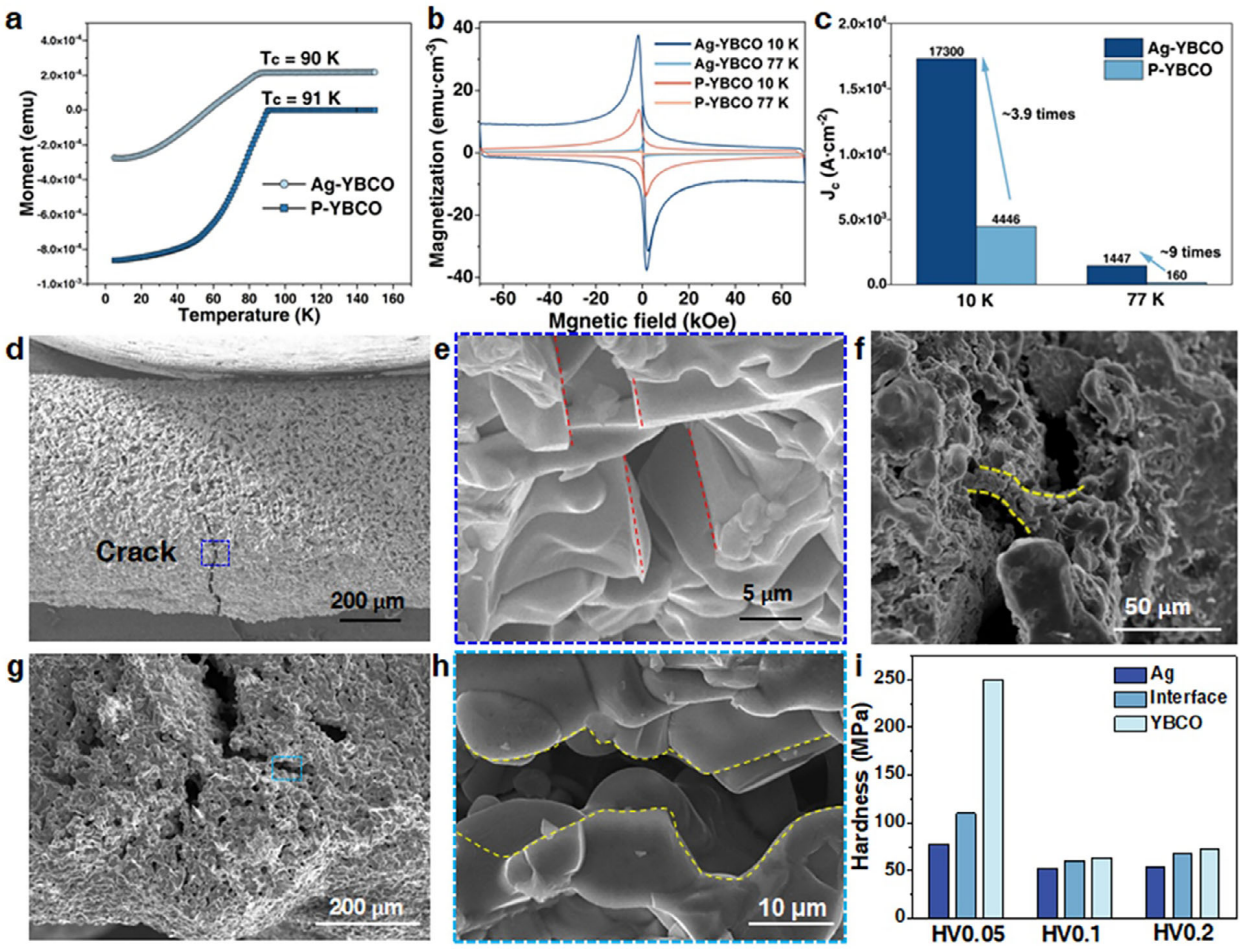
Superconducting and micro‐mechanical characterization of the Ag‐YBCO composite. (a) Magnetization‐temperature (*M‐T*) curves of Ag‐YBCO and P‐YBCO. (b) Magnetization‐field (*M‐H*) curves of Ag‐YBCO and P‐YBCO. (c) Comparison of *J*_c_ between Ag‐YBCO and P‐YBCO at different temperatures. (d) SEM image of an in situ three‐point bending test of Ag‐YBCO. (e) Crack propagation mode in Ag‐YBCO, showing transgranular fracture. (f) Plastic bridging zone of 5–10 µm at the macroscopic YBCO‐Ag interface. (g) SEM image showing the microstructure and crack propagation in the P‐YBCO sample. (h) Crack propagation mode of P‐YBCO showing intergranular fracture. (i) Micro‐Vickers hardness measurements of Ag‐YBCO, showing the order: YBCO > Ag/YBCO interface > Ag.

We evaluated the superconducting properties of both the Ag‐YBCO composite and P‐YBCO by magnetization measurements under a magnetic field applied perpendicular to the sample axis (*H* ∥ *c*), including *T*_c_ and *J*_c_. The Ag‐YBCO composite exhibited a *T*_c_ of 90 K under zero‐field‐cooled (ZFC) conditions, while P‐YBCO showed a *T*_c_ of 91 K (Figure 5a). The close transition temperatures indicate that Ag incorporation does not significantly affect the superconducting transition behavior of the Y123. Figure 5b shows the magnetization as a function of applied magnetic field (*M‐H*) for the two samples. The Ag‐YBCO composite exhibits significantly higher magnetization than P‐YBCO, indicating an enhanced *J*_c_. The width of the magnetization hysteresis loop (Δ*M*) in the perpendicular direction is related to *J*_c_. Using the extended Bean model, the *J*_c_ of the Ag‐YBCO and P‐YBCO samples was calculated and analyzed at different temperatures with the applied field parallel to the sample axis (Section S3 and Figures S19–S21). At 10 K, *J*_c_ of Ag‐YBCO reaches 1.73 × 10^4^ A·cm^−2^, which is significantly higher than that of P‐YBCO (4.45 × 10^3^ A·cm^−2^ at 10 K), representing an enhancement of 3.9 times (Figure 5c). The linear *M‐H* response observed in pure Ag at 10 K (Figure S22) confirms that the superconductivity of the composite structure originates solely from YBCO. When the external magnetic field is applied at different orientations with respect to the textured microstructure, the Ag‐YBCO composite exhibits pronounced anisotropic superconducting behavior. Systematic magnetization measurements were performed with the magnetic field applied at an intermediate angle of 45° relative to the sample long axis (Figure S23a,b), as well as parallel to the *a–b* plane (*H* ∥ *a‐b*; Figure S23c). At 10 K, the *J*_c_ reaches its maximum value when *H* ⟂ *a‐b*, decreases for *H* ∥ *a‐b*, and exhibits an intermediate value of 5.77×10^3^ A·cm^−2^ at 45° (Figure S23d). This angular dependence of *J*_c_ reflects the strong crystallographic texture induced by solid‐solution‐driven orientation control, further confirming that the macroscopic superconducting performance is closely correlated with the texture orientation of the Ag‐YBCO composite. This behavior is consistent with that of directionally solidified samples reported in the literature [50], confirming pronounced texture‐ and orientation‐dependent effects.

To quantitatively evaluate the relative contributions of texture formation, grain refinement, Y211 flux pinning, and Ag‐induced grain‐boundary bridging to the *J*_c_, we designed a series of control samples and measured their *M–H* curves (Figure S24a). These include freeze‐dried pure YBCO (P‐YBCO FD), naturally dried YBCO with 10 wt.% Y211 addition (YBCO + 10% Y211 ND), naturally dried Ag‐YBCO (Ag‐YBCO ND), and liquid‐nitrogen‐quenched Ag‐YBCO prepared via freeze drying (Ag‐YBCO LN FD), with naturally dried pure YBCO (P‐YBCO ND) serving as the reference. At 10 K and 1000 Gs, *J*_c_ follows the order (Figure S24b,c): freeze‐dried Ag‐YBCO (Ag‐YBCO FD; optimized process) > Ag‐YBCO ND > Ag‐YBCO LN FD > YBCO + 10% Y211 ND > P‐YBCO FD > P‐YBCO ND. The introduction of pore structures via freeze drying yields a limited enhancement, with Δ*J*_c_^pore^ = *J*_c_(P‐YBCO FD) − *J*_c_(P‐YBCO ND) = 1147 A·cm^−2^, indicating that porosity alone plays a minor role, mainly by facilitating mass transport. The YBCO + 10% Y211 ND sample shows a moderate enhancement (Δ*J*_c_^211^ = 1508 A·cm^−2^) relative to P‐YBCO ND, indicating that Y211 inclusions enhance *J*_c_ via flux pinning, though their contribution alone is insufficient for substantial performance improvement. In contrast, Ag‐YBCO ND exhibits a substantially larger enhancement (Δ*J*_c_^GB^ = 2698 A·cm^−2^), highlighting the dominant role of Ag‐induced grain‐boundary bridging in improving intergranular current connectivity. Although Ag‐YBCO LN FD shows refined grains (∼1.11 µm) (Figure S24d), its lower Δ*J*_c_^GS^ (≈ 1586 A·cm^−2^) indicates that grain refinement alone is not decisive and is strongly constrained by pore connectivity and Ag distribution. Overall, the Ag‐YBCO FD sample achieves a synergistic optimization of pore architecture, Ag bridging, and grain size, resulting in a pronounced enhancement of *J*_c_ and underscoring the importance of microstructural synergy.

To examine the influence of Ag incorporation on the fracture behavior of YBCO, in situ SEM three‐point bending tests were performed (Video S1). The addition of Ag markedly changes the fracture mode of YBCO (Figure 5d,e). Cracks in the Ag‐composite sample propagate transgranularly through the YBCO grains, resulting in flat and sharp fracture surfaces (Figure S25a,b). This indicates that Ag enhances intragranular bonding strength and promotes crack penetration across grains rather than along grain boundaries. The fracture morphology (Figure 5f) reveals a 5–10 µm plastic bonding zone was observed at the Ag‐YBCO interface. In contrast, the Ag‐free P–YBCO sample exhibits intergranular fracture (Figure 5g,h; Figure S25c,d), where cracks propagate along weak grain boundaries, resulting in a tortuous and uneven fracture surface. Micro‐Vickers hardness tests on the Ag‐YBCO composite under different loads (Figure 5i; Figure S26 and Table S7) revealed a distinct gradient in microhardness: YBCO possessed the highest hardness, followed by the Ag/YBCO interface, with Ag being the softest. Additionally, nanoindentation tests performed at different indentation depths (400, 800, and 1200 nm; Figure S27) reveal that Ag exhibits the highest hardness and elastic modulus, YBCO follows, and the interface region shows the lowest mechanical performance. High‐resolution SEM observations of the Ag‐YBCO interface show that the interface is generally dense and well bonded, without discernible macroscopic pores or interfacial cracks (Figure S28a). Nevertheless, careful examination reveals the presence of sparse, locally distributed elongated nanoscale pores at the interface (Figure S28b,c). These pores typically exhibit lengths on the order of ∼1 µm and widths in the nanometer range, and are preferentially aligned along the interfacial region. The reduced mechanical properties measured in the interfacial region can be reasonably correlated with these nanoscale pores. Although the Ag–YBCO interface is predominantly dense, the presence of elongated nanoscale pores locally decreases the effective load‐bearing area and acts as stress concentration sites during nanoindentation, leading to a lower measured hardness and elastic modulus compared with the bulk YBCO and Ag phases. It should be noted that this behavior reflects the local nanoscale mechanical response of the interfacial region and does not contradict the overall structural integrity and tight bonding observed at the Ag–YBCO interface.

## 3 Discussion and Outlook

The investigation of dual‐material composite systems often requires the construction of compositional gradient structures to gain deeper insights into the interactions between different components [51, 52, 53, 54]. Conventional thermal diffusion methods typically achieve gradient control by mixing two powders in varying ratios [36, 37, 55]. However, these approaches are not only time‐consuming and complex but also highly sensitive to minor deviations in stoichiometry, which can obscure or cause key mechanistic insights to be missed. This study employs a dual‐material co‐extrusion technique to fabricate a macroscopic Ag‐YBCO composite. A radially aligned porous structure was created on the water‐based YBCO side through freeze‐drying. This architecture serves a dual purpose: first, the pores provide pathways for the directional diffusion of Ag^+^ ions along the channels during the high‐temperature reaction, thereby spontaneously forming a continuous and controllable composition gradient. This offers an ideal model for systematically studying the diffusion and reaction behavior between the two materials. Second, the aligned porous structure facilitates the characterization and observation of interface evolution and microstructural changes along both sides of the pores. Utilizing this method, we systematically elucidated the coupling relationships among solute diffusion, phase formation, and oriented growth within the Ag‐YBCO system.

Solution‐driven texturing typically occurs during the recrystallization of alloys [56, 57, 58]. Its underlying mechanism involves the interaction between solute atoms and crystal defects, which governs the evolution of a microstructure with strong preferred orientation [59]. In the Ag‐YBCO system studied here, however, we discovered a solution‐driven texturing mechanism distinct from that in conventional alloy systems. Specifically, the introduction of Ag^+^ solute atoms alters the local structural environment of the Y123 lattice, inducing a slight expansion along the *c*‐axis. This promotes the formation of crystal planes with lower surface energy in the [001] direction, leading to the generation of a YBCAO solid solution phase with orientational characteristics. The favorable coherent matching relationship between YBCAO and the Y123 lattice facilitates the transmission of orientation information through atomic‐scale structural continuity. This, in turn, induces subsequently formed Y123 grains to inherit the same [001] preferred orientation. Unlike the texture formed via the recrystallization‐solute drag effect in traditional alloys, this texturing process does not rely on grain boundary migration or solute segregation. Instead, it is driven by a solute‐lattice interaction‐dominated process of directional structural relaxation and orientated growth.

Currently, the texturing of YBCO materials primarily relies on three categories of methods: chemical deposition [60], melt‐textured growth [61], and seed crystal induction [62]. The first approach typically involves creating a biaxially oriented buffer layer on a substrate to enable the epitaxial growth and orientation control of YBCO thin films. This method can achieve excellent texture consistency and exceptionally high critical current density, making it the core fabrication process for second‐generation high‐temperature superconducting tapes [63]. However, it suffers from a complex preparation procedure, high equipment costs, and limitations imposed by the substrate material and dimensions. Melt texture growth induces grain orientation along the *c*‐axis through a partial melting‐recrystallization process. This approach is suitable for manufacturing bulk materials and large‐sized superconducting components. Nevertheless, the texture uniformity in this method is susceptible to factors such as cooling rate, temperature gradient, and nucleus distribution, often leading to unstable microstructural orientation [64]. The seed crystal induction technique involves introducing well‐oriented seed crystals into the matrix to promote the growth of surrounding grains along the same orientation, thereby achieving directional texture in large bulk materials [65]. However, it still depends on precise thermal treatment and meticulous seed crystal placement [66]. In contrast, the novel solution‐driven texturing mechanism proposed in this study requires neither biaxially oriented buffer layer, seed crystals, nor precise thermal gradients. Instead, it utilizes solute‐lattice interactions to induce lattice orientation relaxation at the atomic scale. Facilitated by the coherent matching between the YBCAO solid solution phase and the Y123 lattice, it promotes the growth of grains with aligned orientation. This mechanism demonstrates that in cooper‐oxide superconductors, solid solution doping can similarly achieve macroscopic texture formation through atomic‐level structural modulation.

In summary, this study addresses a long‐standing key issue in applied superconductor research: although Ag doping significantly enhances the overall performance of YBCO, the underlying mechanism has remained unclear. Through innovative structural design, our work overcomes the limitations of traditional doping methods, uncovers a new mode of solid‐solution‐driven texturing, and fundamentally elucidates the intrinsic reasons for the improvements in both superconductivity and mechanical properties:

- We present an innovative methodology for investigating interaction mechanisms in composite materials. This approach involves constructing a macroscopic Ag‐YBCO composite via dual‐material co‐extrusion and introducing directional pores into the YBCO through freeze casting. During annealing, Ag^+^ ions diffuse along these channels to form a continuous concentration gradient.  This approach successfully overcomes the limitations of conventional doping strategies, enabling the capture of all key mechanisms triggered by specific compositions.
- We report the first observation of a solid‐solution‐driven texture growth mechanism in YBCO induced by Ag doping.  Partial substitution of Ag atoms into the Y123 lattice leads to the formation of an YBCAO solid‐solution phase that is oriented along the [001] direction. Owing to the coherent interface between YBCAO and Y123, this orientation relationship can be transmitted at the atomic scale to subsequently grown Y123 grains, resulting in a preferred orientation along [001] and promoting macroscopic texturing.
- Ag doping improves intergranular bridging and network formation, while also altering the crack propagation mode within the superconducting grains. Specifically, Ag promotes bridging between Y123 grains, leading to an interconnected network. Mechanically, the presence of Ag modifies the mechanical response of the grain boundaries, shifting the fracture mode from intergranular to transgranular.
(1) $$\gamma =\frac{E_{slab}-n_{salb}E_{bulk}}{2A}\;$$
(2) $$\delta =\frac{dAg_{x}\left(014\right)-dY123\left(104\right)}{dY123\left(104\right)}=0.68\%$$

## 4 Methods

### 4.1 Slurry Preparation

YBCO precursor powder synthesis: Y_2_O_3_ (99.99%, Macklin), CuO (99.5%, Macklin), and BaCO_3_ (99.95%, Macklin) were mixed in a stoichiometric molar ratio (Y: Ba: Cu = 1:2:3) with anhydrous ethanol and ball‐milled using a planetary mill (MSK‐SFM‐1). The mixture was then dried at 90°C for 12 h in an oven to obtain YBCO precursor powder.

Aqueous binder solution formulation: sodium carboxymethyl cellulose (CMC, [C_6_H_7_O_2_(OH)_2_OCH_2_COONa]_n_; viscosity: 600–1000 mPa·s; Macklin) was dissolved in deionized water at 6.5 wt.% under mechanical stirring for 2 h to serve as the binder.

Oil‐based binder solution formulation: ethyl cellulose (EC, (C_12_H_22_O_5_)_n_; viscosity: 300 mPa·s; Macklin) was dissolved in diethylene glycol monobutyl ether (C_8_H_18_O_3_, 99%, Macklin) at 5 wt.%, under mechanical stirring for 2 h at 60°C in a water bath.

The water‐based YBCO slurry was prepared by mixing 50 g YBCO precursor powder with 15 mL of an aqueous binder solution, followed by the addition of 5 g Epoxidized soybean oil (ESO, C_57_H_98_O_18_, Aladdin) and homogenization through roller milling (3 cycles).

The oil‐based Ag slurry was prepared by combining 50 g of Ag nanoparticles (200 nm, 99.99%, Macklin) with 20 mL oil‐based binder solution and 4 g of glycerol (C_3_H_8_O_3_, 99%, Macklin), then homogenized via roller milling (3 cycles).

### 4.2 Fabrication of a Sample with a Macroscopic Ag‐YBCO Interface via Co‐Extrusion

The slit width of the twin‐roll extruder was precisely adjusted to 5 mm. Aqueous YBCO slurry and oil‐based Ag slurry were injected into separate feed tanks, with their flow rates controlled by peristaltic pumps at 5 and 8 mL/min, respectively. The twin rolls were then set to rotate synchronously at 10 rpm, enabling the two slurries to merge steadily at the extrusion die and directly form a composite green body with a continuous and parallel Ag–YBCO interface.

### 4.3 Material and Structure Characterization

X‐ray diffraction (XRD) analysis was performed using an X' Pert PRO MPD diffractometer (PANalytical, Netherlands) with Cu *Kα* radiation over a 2*θ* range of 5°–90°. Microstructural characterization was conducted using field‐emission scanning electron microscopy (FE‐SEM, Apreo S, Thermo Fisher Scientific, USA) equipped with energy‐dispersive X‐ray spectroscopy (EDS, Octane Pro, AMETEK, USA) for elemental mapping at a working distance of 10 mm and accelerating voltage of 15 kV.  Transmission electron microscopy (TEM, Talos F200s, Thermo Fisher Scientific, USA) coupled with a Super‐X EDS system (Bruker, Germany) was employed for nanoscale compositional analysis. Crystallographic orientation analysis was performed via electron backscatter diffraction (EBSD, Symmetry S2, Oxford Instruments, UK) operating at 20 kV with a step size of 50 nm and acquisition rate exceeding 300 points per second (pps). Thermogravimetric analysis (TGA) was carried out using a simultaneous TGA/differential scanning calorimetry (DSC) analyzer (TGA/SDTA 851e, Mettler‐Toledo, Switzerland) from 25°C to 1000°C at a heating rate of 2°C min^−1^ under nitrogen atmosphere.

### 4.4 Measurements

Superconducting properties were characterized by SQUID magnetometry (MPMSR2, Quantum Design, USA), including zero‐field‐cooled (ZFC, 50 Oe) magnetization curves and temperature‐dependent hysteresis loops (10, and 77 K, ±70 kOe). In situ SEM three‐point bending tests (Quanta 650 FEG SEM, FEI, USA with Deben Microtest 5kN module, UK). Hardness was evaluated using micro‐Vickers (FM‐700, Future‐Tech, Japan) and nanoindentation (TI‐950, Hysitron, USA with Berkovich diamond tip).

## Funding

National Key Research and Development Program of China grant 2024YFB4607300, National Natural Science Funds for Distinguished Young Scholar grant 12325205, Distinguished Talent Research Funding of Lanzhou grant 127000–563225112, Science and Technology Leading Talent Project of Gansu Province grant 24RCKB008.

## Conflicts of Interest

The authors declare no conflict of interest.

## Supporting information




**Supporting File 1**: advs74354‐sup‐0001‐SuppMat.docx.

**Supporting File 2**: advs74354‐sup‐0002‐VideoS1.mp4.

**Supporting File 3**: advs74354‐sup‐0003‐DateFile.zip.

## Data Availability

All data are available in the main text or the supplementary materials.
