# Supplementary material for: Mechanism of Solid Solution‐Driven Texture Induced by Ag Doping in YBCO Superconductor
Source: Adv Sci (Weinh). 2026 Feb 10;13(22):e22923. doi: 10.1002/advs.202522923 (PMC13088314; doi:10.1002/advs.202522923)
Supplement: Supplementary file 1 — Supporting File 1: advs74354‐sup‐0001‐SuppMat.docx. [file ADVS-13-e22923-s003.docx]

**Supplementary Information**

**Mechanism of solid solution-driven texture induced by Ag doping in YBCO superconductor**

Fenyan Zhao^1,2^†, Baoqiang Zhang^1,2^†, Xiyang Su^3^, Yantang Zhao^1,2^，Xingyi Zhang^1,2^*

**Affiliations:**

^1^Key Laboratory of Mechanics on Disaster and Environment in Western China attached to the Ministry of Education of China, Lanzhou University; Lanzhou, Gansu 730000, PR China

^2^Department of Mechanics and Engineering Sciences, College of Civil Engineering and Mechanics, Lanzhou University; Lanzhou, Gansu 730000, PR China

^3^Department of Mechanical and Aerospace Engineering, The Hong Kong University of Science and Technology; Hong Kong, 999077, PR China.

*Corresponding author. Email: [zhangxingyi@lzu.edu.cn](mailto:zhangxingyi@lzu.edu.cn)

†These authors contributed equally to this work.

**The PDF file includes:**

Supplementary Discussion

Figs. S1 to S28

Tables S1 to S7

References

**Other Supplementary Materials for this manuscript include the following:**

Videos S1

Data S1 to S3

**Supplementary Discussion**

**Section 1: Parameter settings for structural optimization and energy calculations using the CASTEP module in Materials Studio^1-4^**

All calculations were conducted within the framework of the generalized gradient approximation (GGA), using the Perdew-Burke-Ernzerhof (PBE) functional to describe exchange-correlation interactions, and ultrasoft pseudopotentials to represent the interactions between atomic nuclei and valence electrons. The plane-wave basis set included the valence electron configurations of all constituent elements, with a cutoff energy of 800 eV. Brillouin-zone *k*-point sampling was carried out using the Monkhorst–Pack scheme. Different *k*-point grids were employed for different supercell configurations to ensure sufficient sampling accuracy. Specifically, a 3 × 2 × 4 *k*-point mesh was used for the YBa_2_Cu_2.8333_Ag_0.1667_O_7-δ_ (*δ* = 0, 0.5, 1) models, whereas 3 × 3 × 2 meshes were adopted for the YBa_2_Cu_2.85_Ag_0.15_O_6.8_ and YBa_2_Cu_2.8125_Ag_0.1875_O_6.75_ models. The high-symmetry points in the Brillouin zone were defined as: Γ(0,0,0), X(0.5,0,0), Y(0,0.5,0), S(0.5,0.5,0), Z(0,0,0.5), U(0.5,0,0.5), T(0,0.5,0.5), R(0.5,0.5,0.5) and Ś(0.5,0.5,0.25). The electronic band structure was calculated along these high-symmetry paths. For the Mott insulator YBa_2_Cu_3_O_6_, the GGA+U approach was applied with spin-polarized correction for the Cu 3*d* orbitals to accurately account for strong correlation effects, setting *U* = 9 eV. Structural optimizations were carried out using the BFGS algorithm, allowing full relaxation of both lattice parameters and atomic positions. Convergence criteria were set as follows: total energy change < 1×10^-6^ eV/atom, atomic forces < 0.01 eV/Å, and stress tensor components < 0.02 GPa. Self-consistent field (SCF) iterations were considered converged when the total energy change was below 5×10^-6^ eV/atom, with atomic forces and internal pressure converged to within 0.01 eV/Å and 0.05 GPa, respectively.

**Section 2: Analysis of incoherent phase boundaries between YBCAO and Y211**

As revealed in Fig.S26, the interfacial structural relationship between YBCAO(030) and Y211(311) was characterized. Through FFT and IFFT analysis of HRTEM images, the interplanar spacings were measured as 1.302 Å for YBCAO(030) and 2.157 Å for Y211(311), with an interfacial misorientation angle of *θ* ≈ 24°. The lattice mismatch was quantitatively evaluated by the following formula^5^:

$\delta=\frac{dY211\left( 311 \right)\cdot\cos\theta-dYBCAO(030)}{dYBCAO(030)}=51.34\%$ (1)

Since *δ* > 25%^6^, this indicates an incoherent interface between YBCAO(030) and Y211(311).

**Section 3: Derivation of the extended Bean model for perpendicular magnetic fields in Ag-YBCO composite**

Gyorgy et al.^7^ proposed an extended Bean critical-state model for anisotropic YBCO superconductors:

$\Delta M=\frac{J_{c1}t}{20}(1-\frac{t}{3l}\frac{J_{c1}}{J_{c2}})$, (2)

$\frac{J_{c1}}{J_{c2}}<\frac{l}{t}$

Where *J*_c1_ represents the current density parallel to the Cu-O planes (within the *a-b* plane), while *J*_c2_ denotes the current density perpendicular to the Cu-O planes (along the *c*-axis), with the magnetic field oriented normal to the surface of dimensions *l*×*t*. When $l\gg\frac{J_{c1}t}{{3J}_{c2}}$, the system reduces to an infinite strip of thickness *t*, whose solution takes the form:

$\Delta M=\frac{J_{c1}t}{20}$ (9)

We adapt the anisotropic Bean model proposed by Gyorgy et al.^7^ to the geometric characteristics of our Ag-YBCO composites (with the magnetic field perpendicular to the sample axis), introducing appropriate simplifications and modifications to establish a *J*_c_ calculation model applicable to the composite structure investigated in this study. Given the structural characteristics of the composite, the contribution of the radial *J*_c_ to the magnetization hysteresis width (ΔM) can be neglected (Fig.S17). As illustrated in Fig.S13a, the Ag-YBCO composite exhibits a circular cross-section with an Ag radius of *R*_1_ and an overall composite radius of *R*_2_. The *M-H* curve of pure Ag at 10 K (Fig.S16) displays a linear response, confirming that the superconductivity of the composite originates entirely from YBCO. Consequently, in the extended Bean model calculations, Ag contributes only to the geometric parameters and does not participate in the determination of *J*_c_. The YBCO shell forms a hollow cylindrical structure (Fig.S13b) with a radial width of *R*_2_ − *R*_1_. To facilitate modeling, this hollow cylinder can be equivalently represented as two superimposed rectangular strips (Fig.S13c), each with thickness *t* and width *R*_2_ − *R*_1_, such that their combined cross-sectional area matches that of the hollow cylinder. The expression for the equivalent thickness *t* is given by:

$t=\frac{\pi{R_{2}}^{2}-\pi{R_{1}}^{2}}{2(R_{2}-R_{1})}=\frac{\pi}{2}(R_{2}+R_{1})$ (10)

Based on this, the formula for calculating *J*_c_ in the Ag-YBCO composite structure using the extended Bean model is:

$J_{c}=\frac{20\Delta M}{t}$,

$t=\frac{\pi}{2}(R_{2}+R_{1})$ (11)

Similarly, by modeling the P-YBCO as a square-cross-section prism (Fig.S14), the expression for *t*is given by:

$t=\sqrt{\pi R^{2}}$ (12)


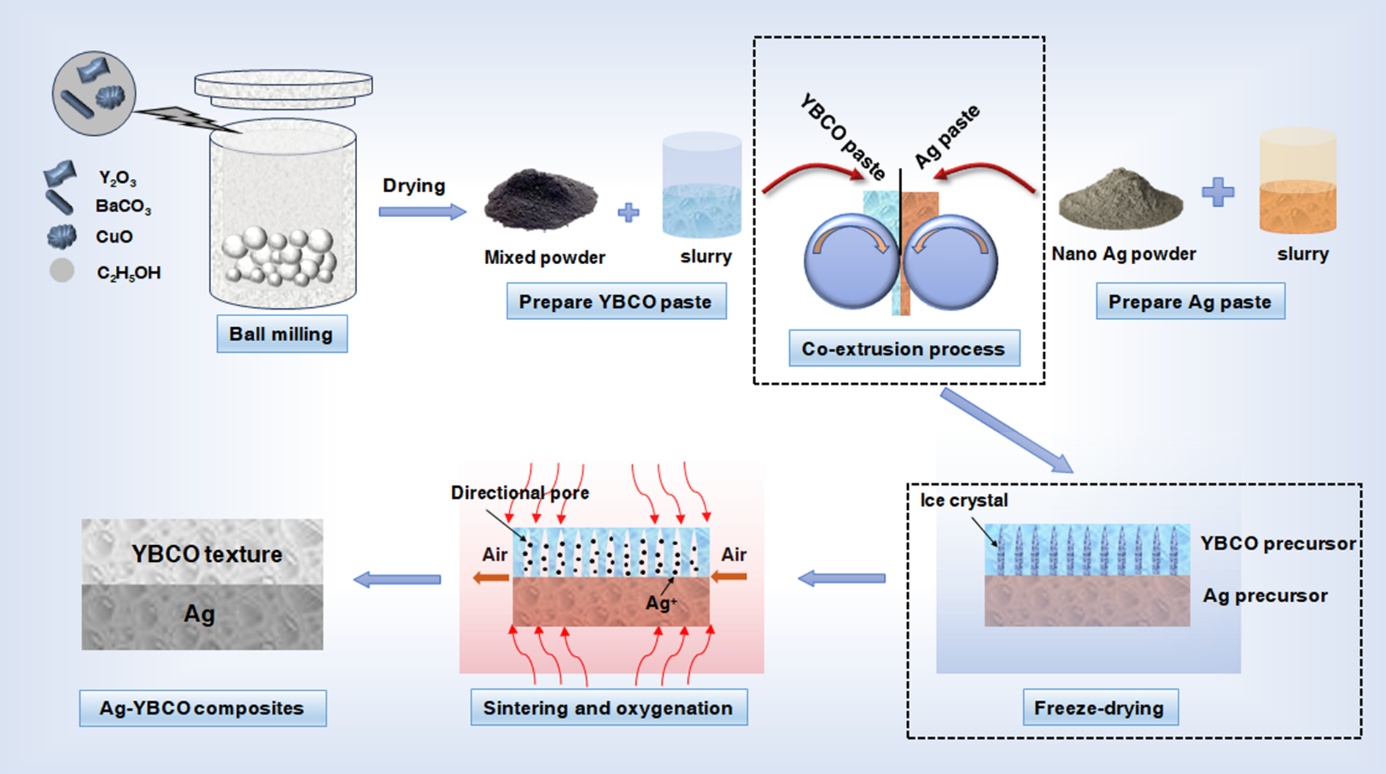


**Figure S1.** Process flow diagram of the Ag-YBCO composite fabricated by dual-material co-extrusion combined with low-temperature cold casting.


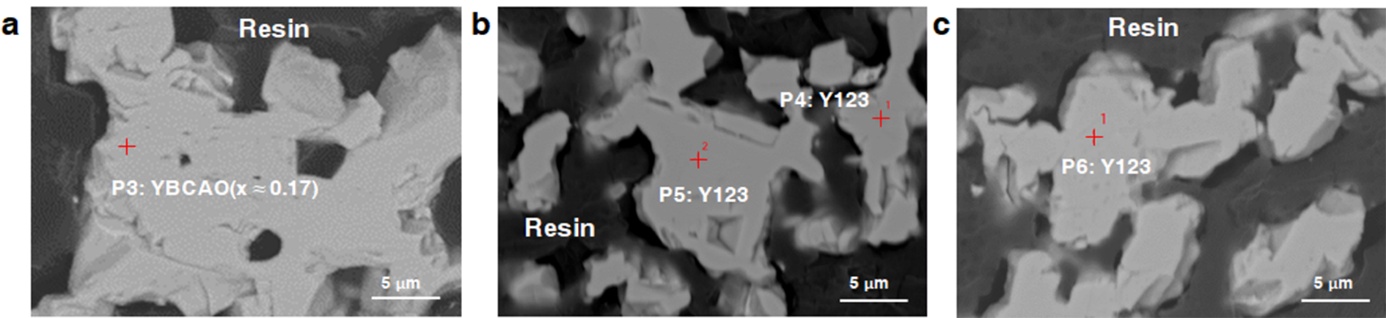


**Figure S2.** EPMA point analysis of the YBCO region. (a) Position P3. (b) Positions P4 and P5. (c) Position P6.


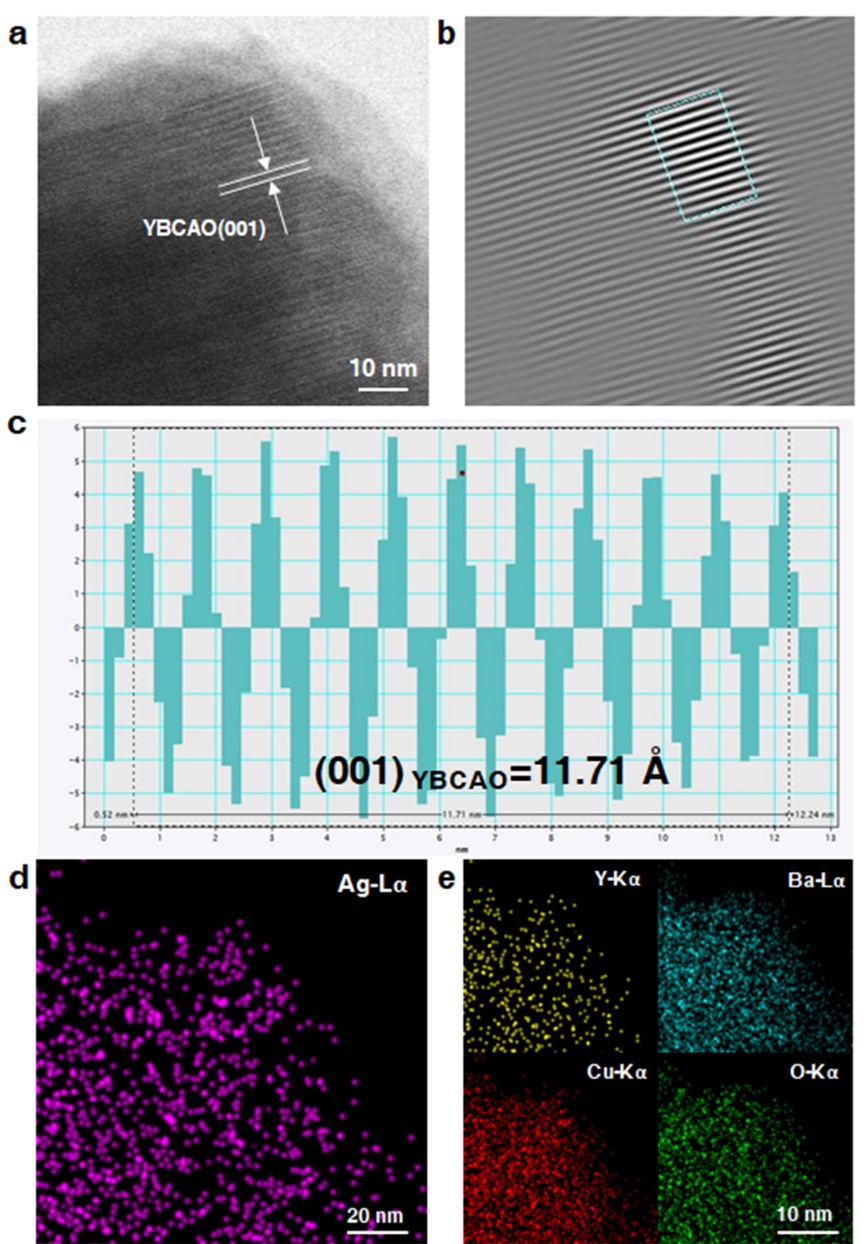


**Figure S3.** BF-STEM characterization of YBCAO(001). (a) BF-STEM image. (b) IFFT image. (c) line profile extracted from the IFFT. (d) STEM–EDS elemental mapping of Ag. (e) STEM–EDS elemental mappings of Y, Ba, Cu, and O.


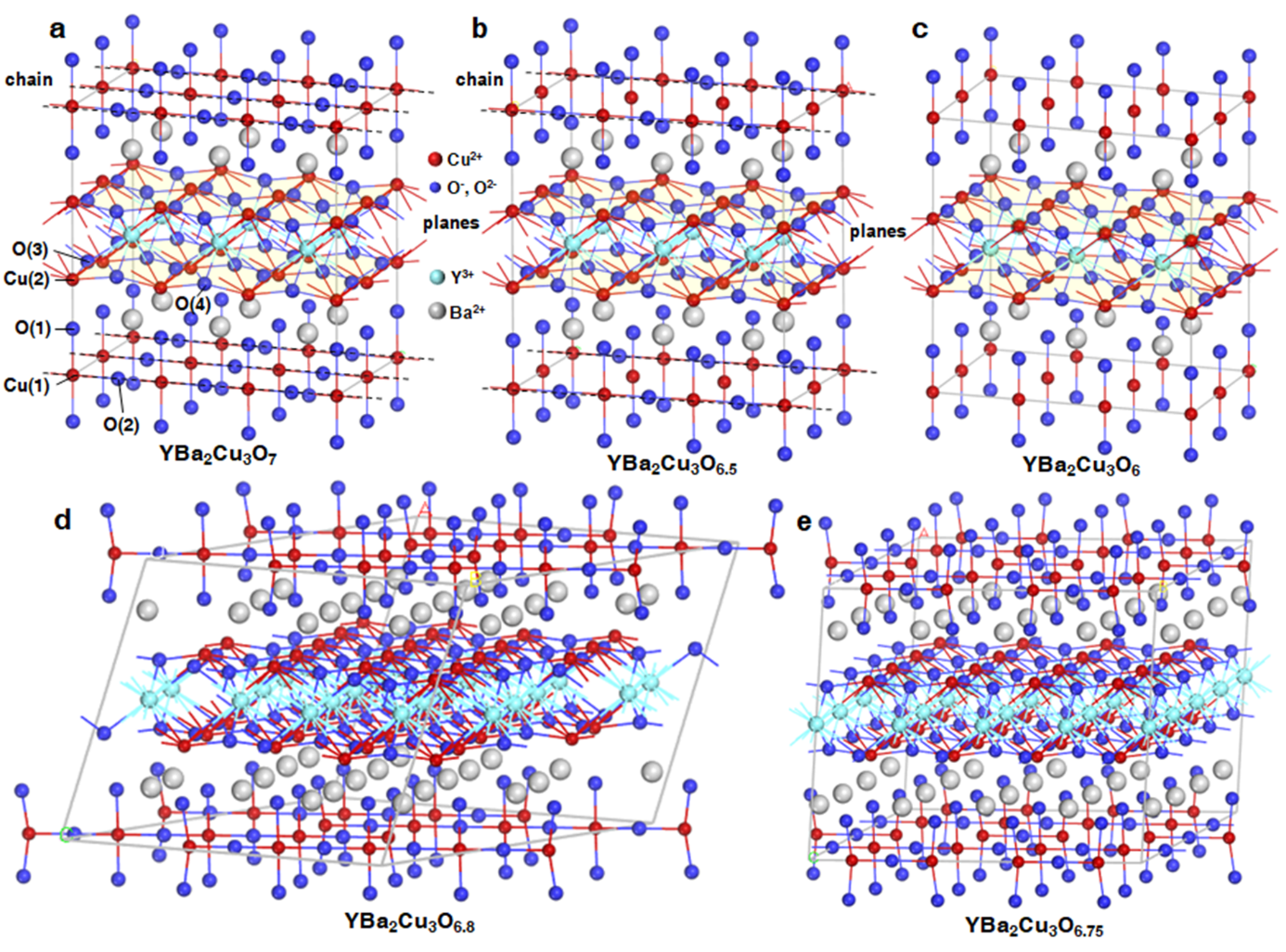


**Figure S4.** Supercell configurations of YBa_2_Cu_3_O_7−δ_ with different oxygen contents and vacancy arrangements. (a–c) Idealized ordered supercell structures of YBa_2_Cu_3_O_7−δ_ with δ = 0, 0.5, and 1, respectively, representing fully oxygenated, moderately oxygen-deficient, and highly oxygen-deficient states, and illustrating the corresponding evolution of the Cu–O chain framework. (d,e) Supercell models of YBa_2_Cu_3_O_6.8_ and YBa_2_Cu_3_O_6.75_, respectively, constructed to approximate experimentally relevant, partially disordered oxygen-vacancy distributions. These models preserve overall oxygen contents within the experimental range while introducing distinct local vacancy configurations, including chain-end and in-chain oxygen vacancies.


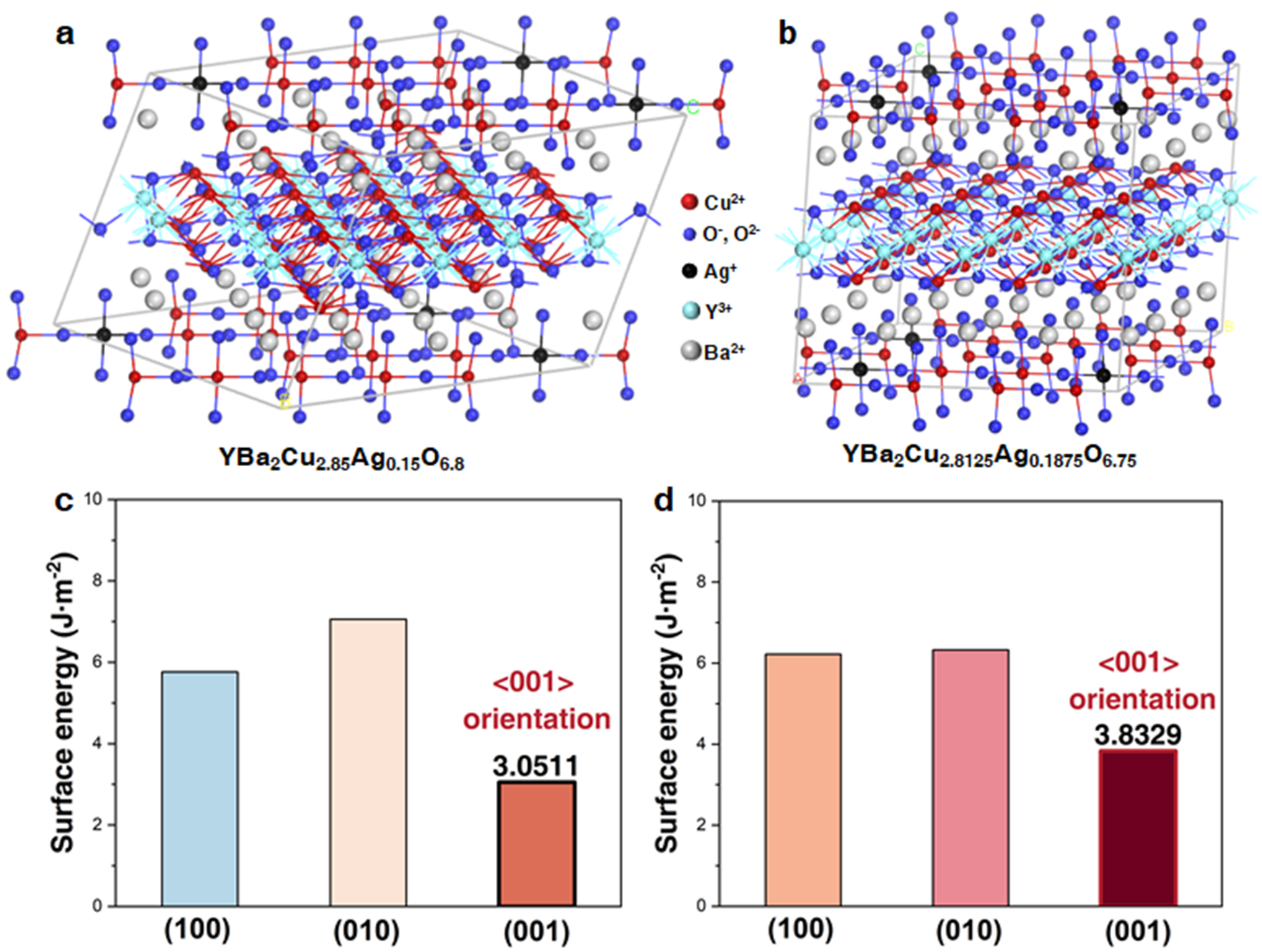


**Figure S5.** Optimized crystal structures and surface energy calculations of Ag-doped YBa_2_Cu_3_O_7−δ_ systems with non-integer oxygen contents. (a,b) Fully relaxed supercell configurations of YBa_2_Cu_2.85_Ag_0.15_O_6.8_ and YBa_2_Cu_2.8125_Ag_0.1875_O_6.75_, respectively. (c,d) Calculated surface energies of the three low-index crystallographic planes, (100), (010), and (001), for YBa_2_Cu_2.85_Ag_0.15_O_6.8_ and YBa_2_Cu_2.8125_Ag_0.1875_O_6.75_, respectively.


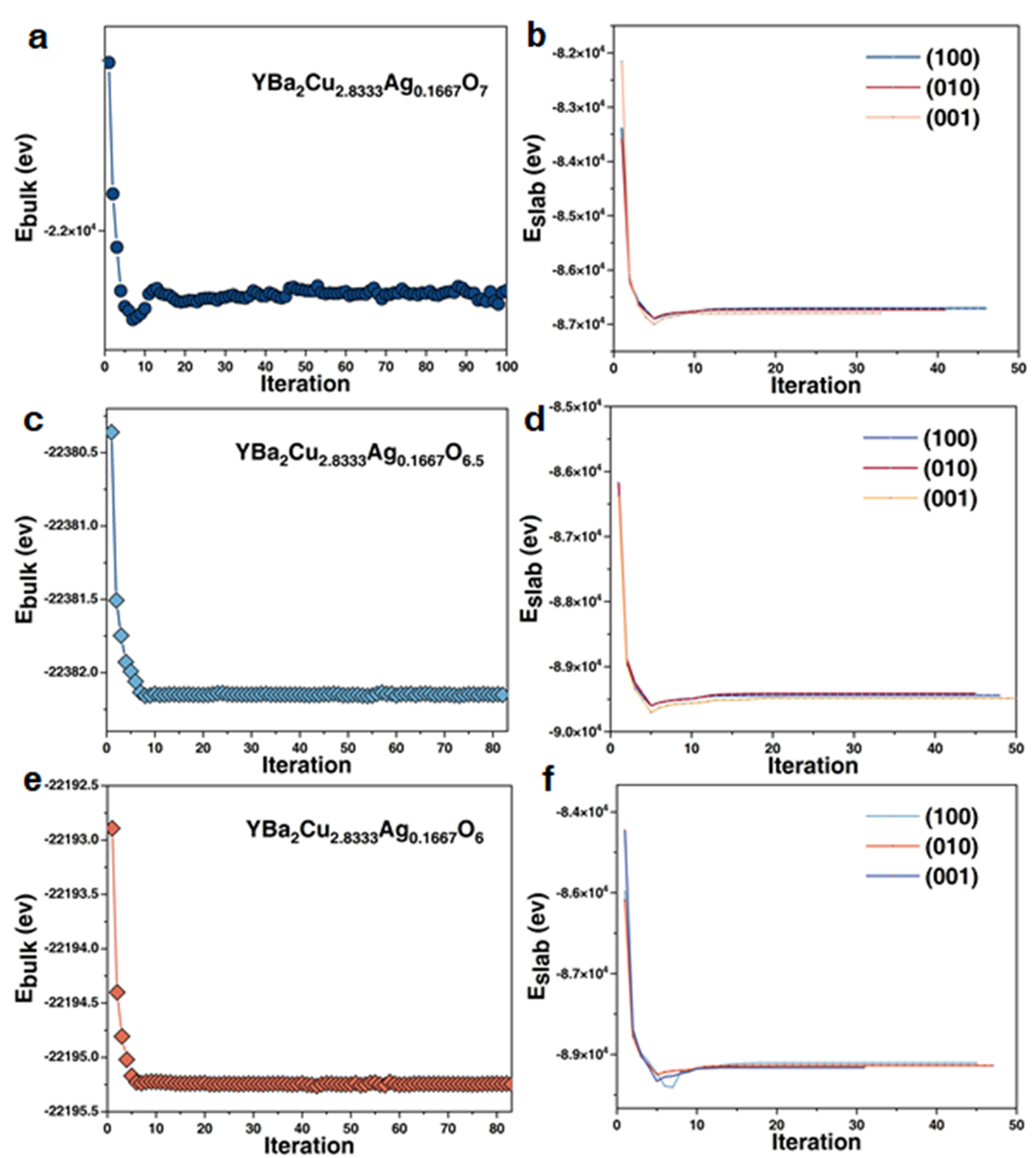


**Figure S6.** First-principles–calculated bulk energies (*E*_bulk_) and surface slab energies (*E*_slab_) of YBa_2_Ag_0.1667_Cu_2.8333_O_7-δ_ with δ = 0, 0.5, and 1. (a,b) *E_bulk_* and *E_slab_* for the (100), (010), and (001) surface models of YBa_2_Ag_0.1667_Cu_2.8333_O_7_. (c,d) *E_bulk_ and E_slab_* for YBa_2_Ag_0.1667_Cu_2.8333_O_6.5_. (e,f) *E_bulk_ and E_slab_* for YBa_2_Ag_0.1667_Cu_2.8333_O_6_.


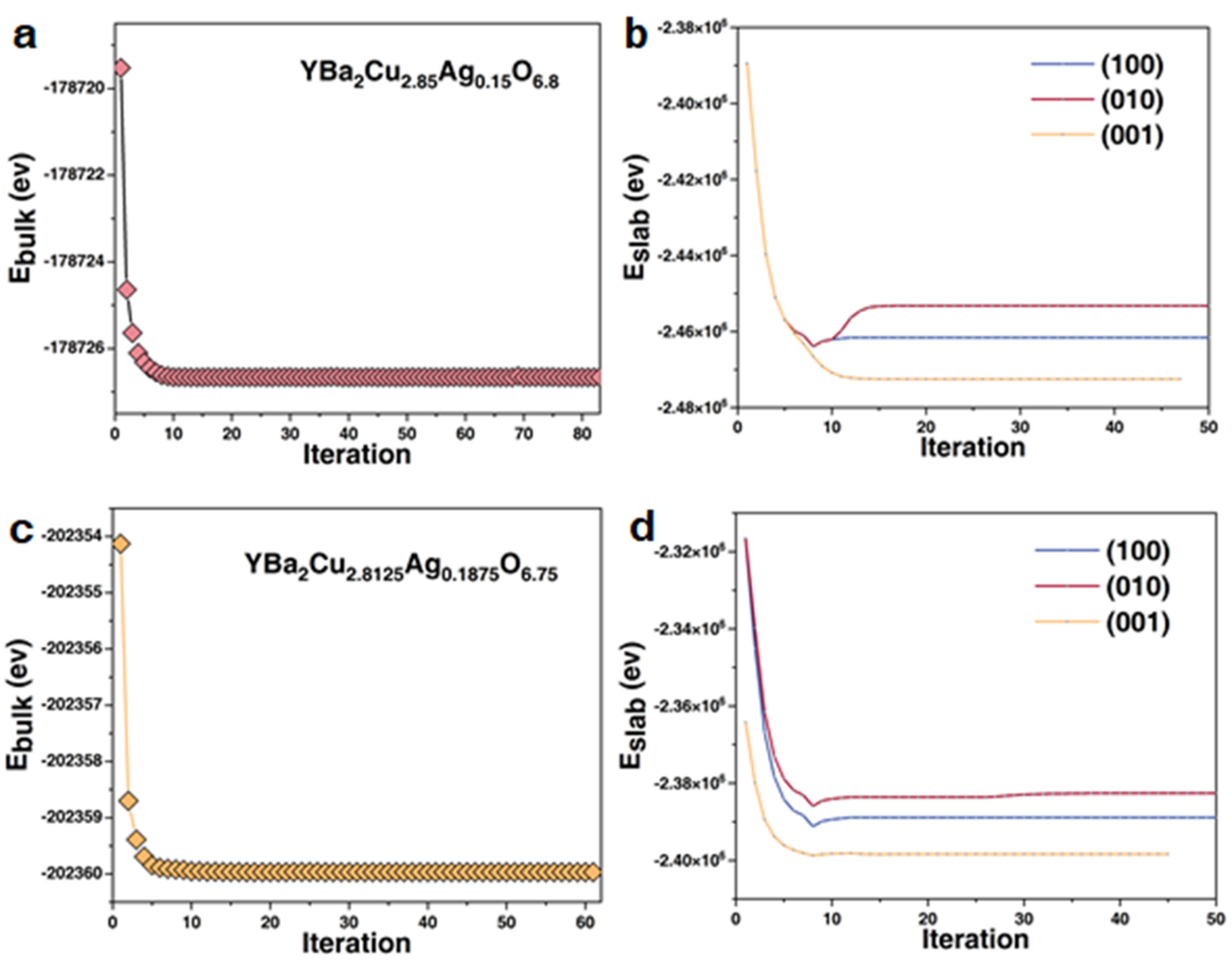


**Figure S7.** First-principles–calculated *E*_bulk_ and *E*_slab_ of YBa_2_Cu_2.85_Ag_0.15_O_6.8_ and YBa_2_Cu_2.8125_Ag_0.1875_O_6.75_. (a) *E_bulk_* and *E_slab_* for the (100), (010), and (001) surface models of YBa_2_Cu_2.85_Ag_0.15_O_6.8_. (b) *E_bulk_ and E_slab_* for YBa_2_Cu_2.8125_Ag_0.1875_O_6.75_.


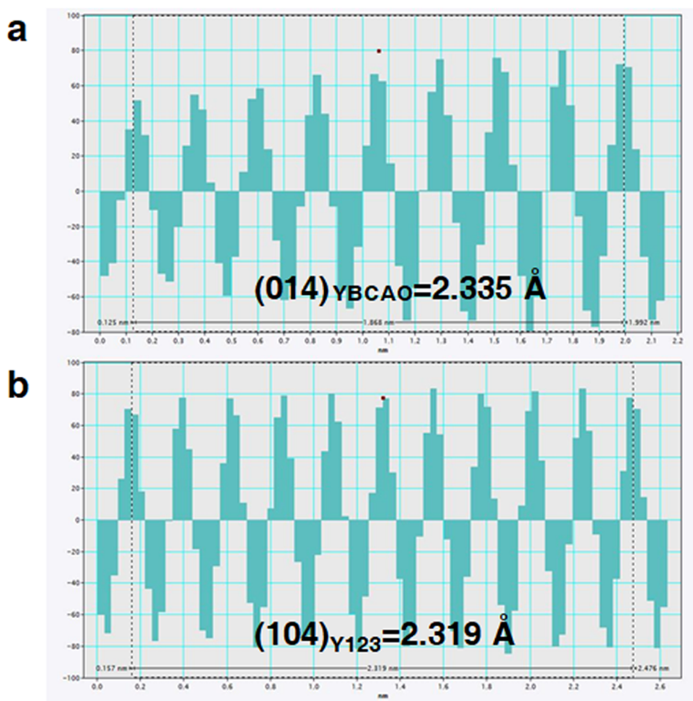


**Figure S8.** IFFT live profile analysis. (a) YBCAO(014). (b) Y123(104).


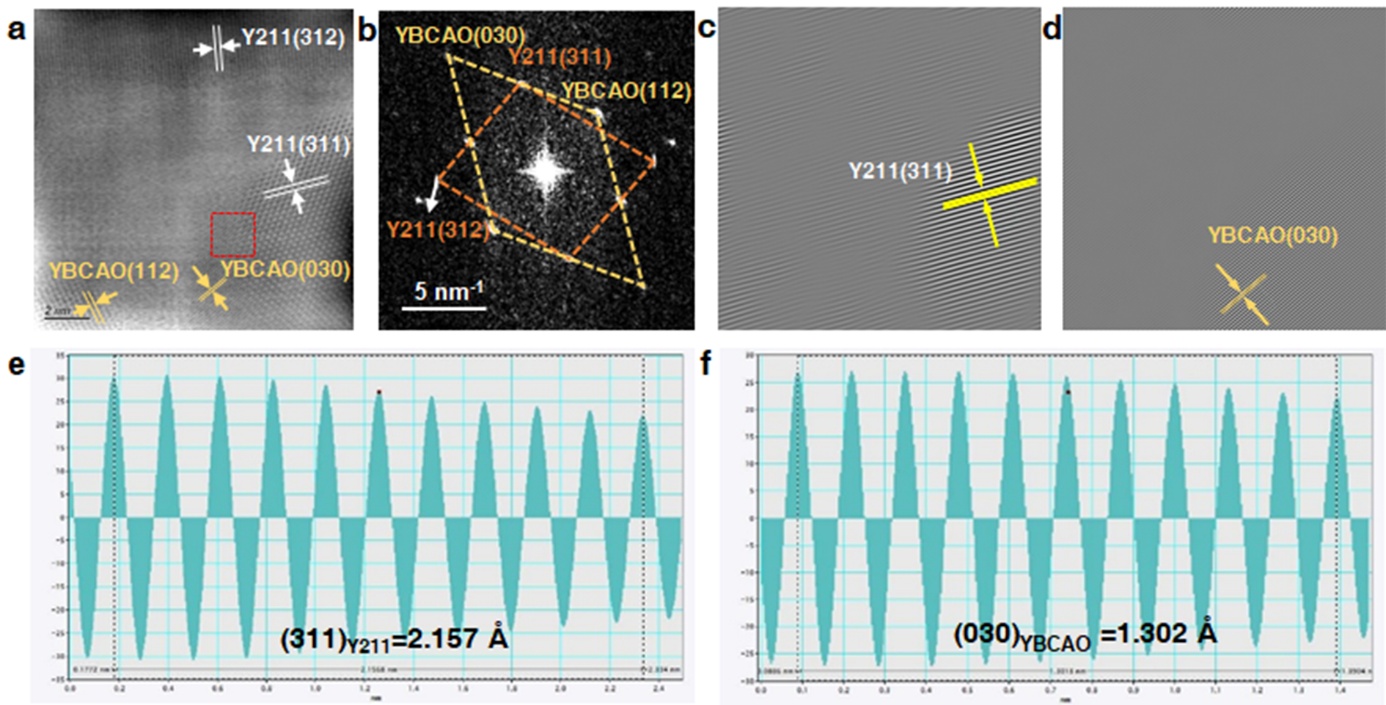


**Figure S9.** Structural characterization of the incoherent Y211(311)/YBCAO(030) interface. (a) HRTEM images. (b) FFT pattern. (c,d) IFFT reconstructions. (e,f) IFFT live profile analysis.


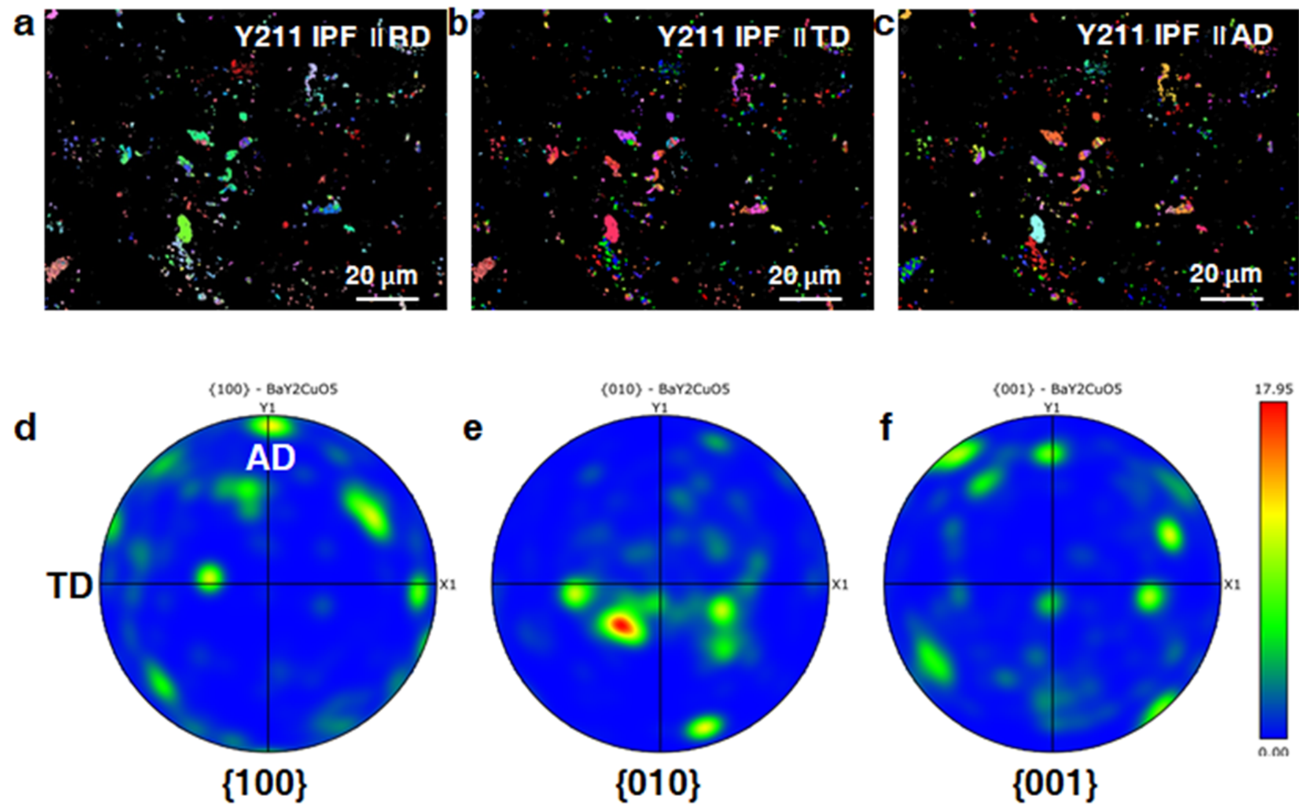


**Figure S10.** Grain orientation analysis of the Y211 phase. (a-c) IPF maps. (d-f) PF.


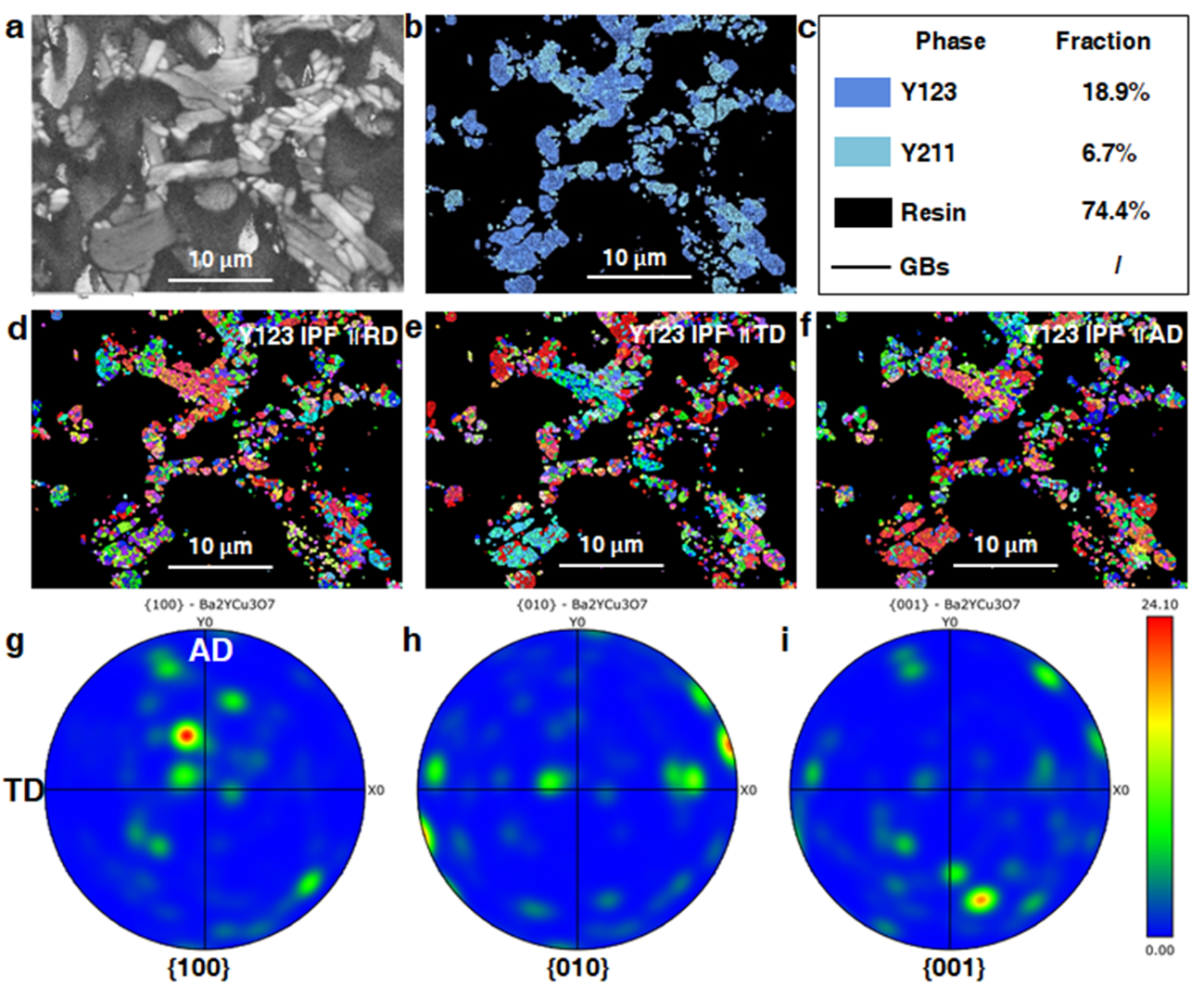


**Figure S11.** Grain orientation analysis of P-YBCO. (a) EBSD band contrast map. (b) Phase distribution map. (c) Color legend and phase fractions corresponding to the phase distribution map. (d-f) IPF maps showing no obvious preferential orientation. (g-i) PF.


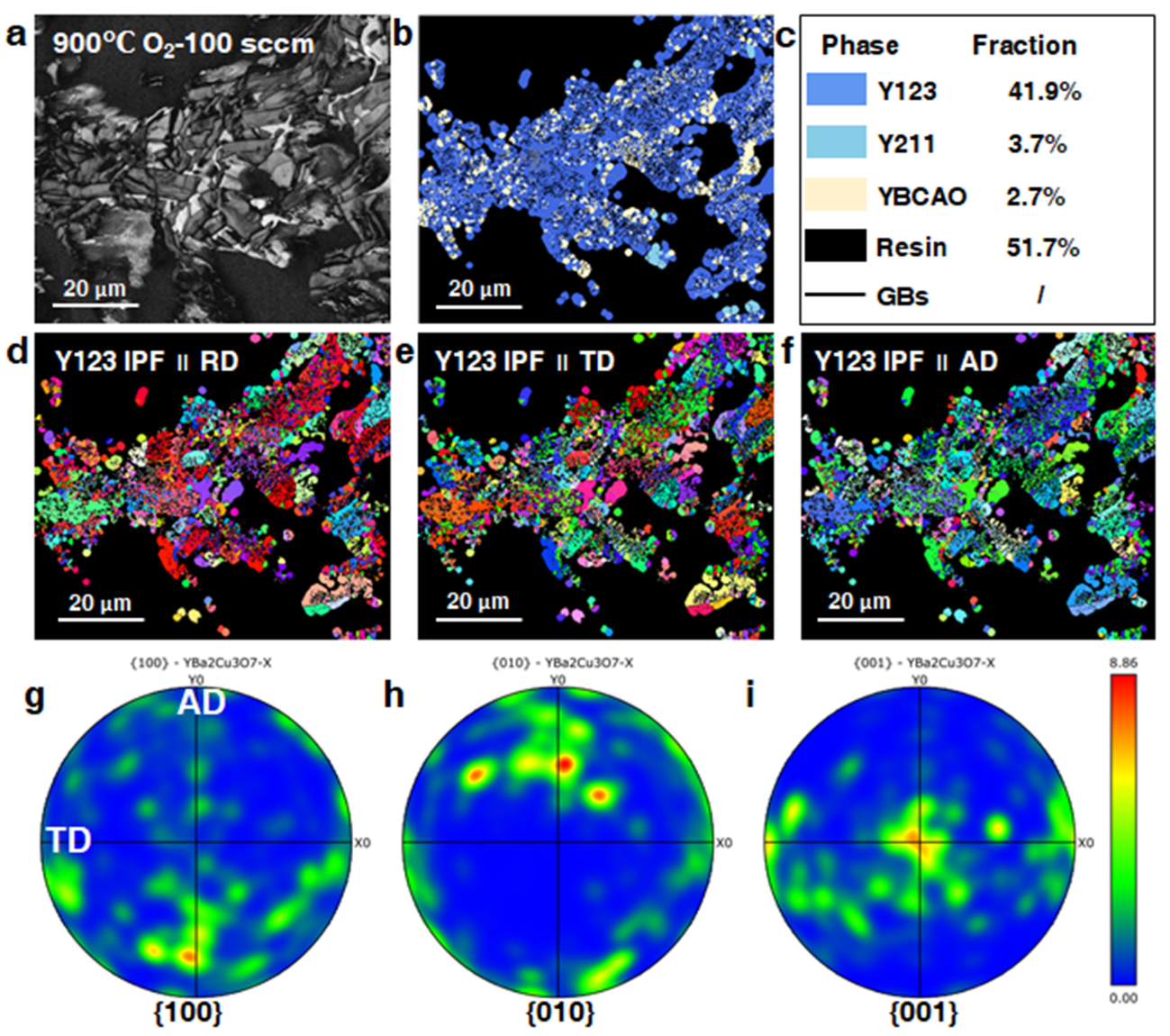


**Figure S12.** EBSD characterization of the Ag–YBCO composite sintered under off-optimal conditions (900 °C, O_2_ flow rate of 100 sccm). (a) Band contrast map. (b) Phase distribution map. (c) Corresponding phase fraction statistics, indicating an increased Y123 fraction (41.9%) and a reduced YBCAO fraction (2.7%) compared with the optimal sintering condition (920 °C in air). (d-f) IPF maps of the Y123 phase along RD, TD, and AD, respectively, showing the presence of [001]-oriented grains along RD but with noticeably weakened orientation concentration. (g-i) PFs of the Y123 phase for the {100}, {010}, and {001} planes, respectively.


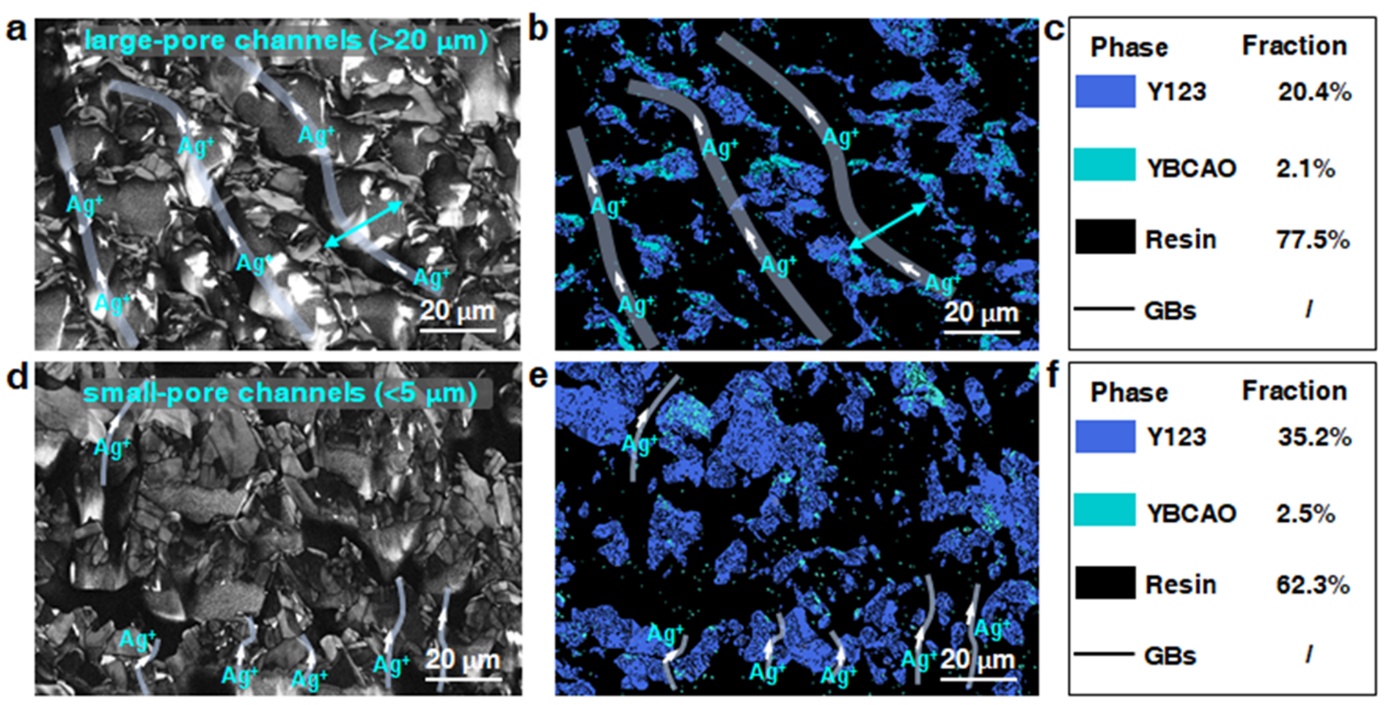


**Figure S13.** EBSD band contrast maps and corresponding phase distribution of samples with different pore sizes formed under slow-freezing and fast-freezing conditions. (a) Band contrast map of the slow-freezing sample with large pores. (b) Corresponding phase distribution map of the slow-freezing large-pore sample. (c) Color legend and quantitative phase fraction statistics associated with (b). (d) Band contrast map of the fast-freezing sample with small pores. (e) Corresponding phase distribution map of the fast-freezing small-pore sample. (f) Color legend and quantitative phase fraction statistics associated with (e).


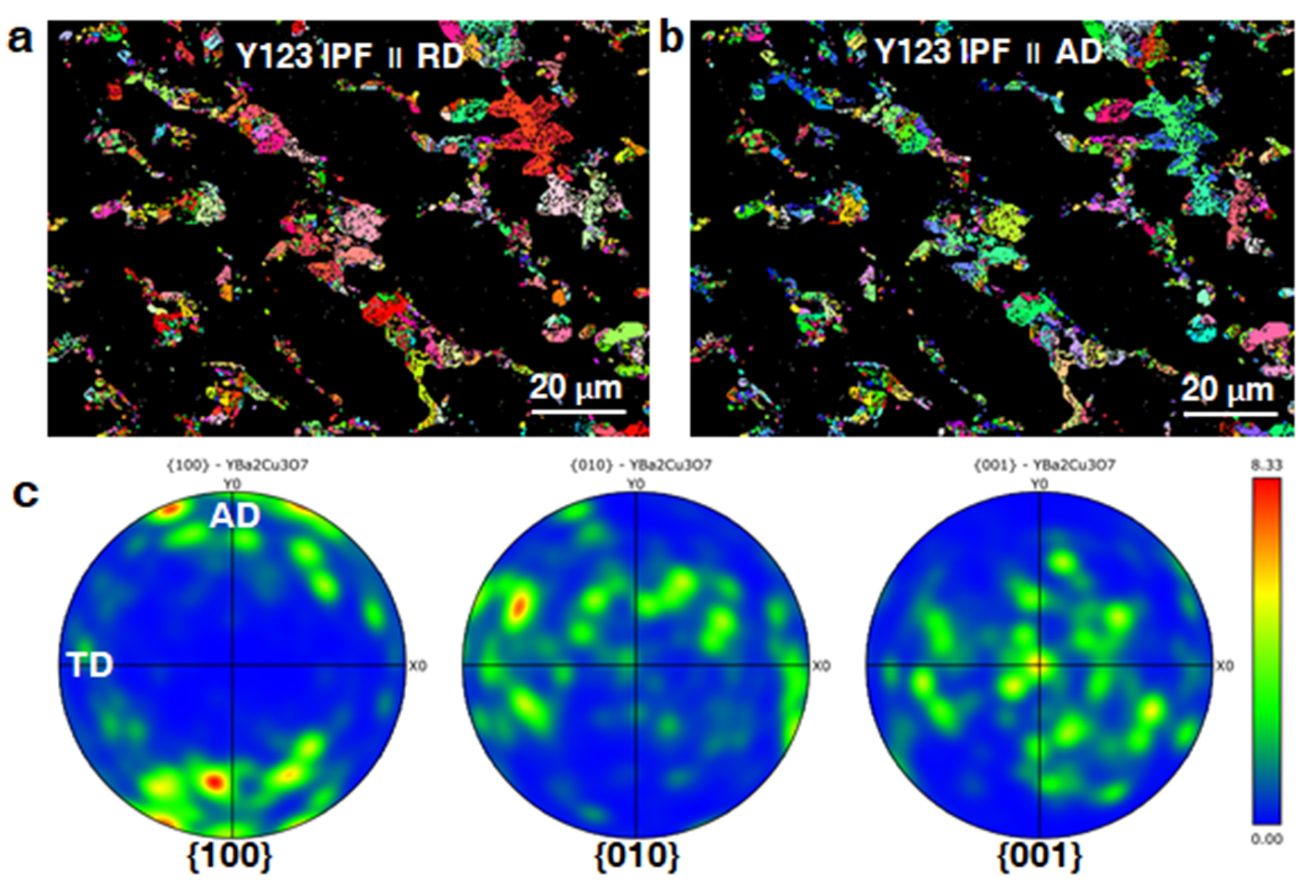


**Figure S14.** EBSD characterization of the large-pore structure generated under slow-freezing conditions. (a) IPF map along RD. (b) IPF map along AD. (c) PF of the {100}, {010}, and {001} crystallographic planes.


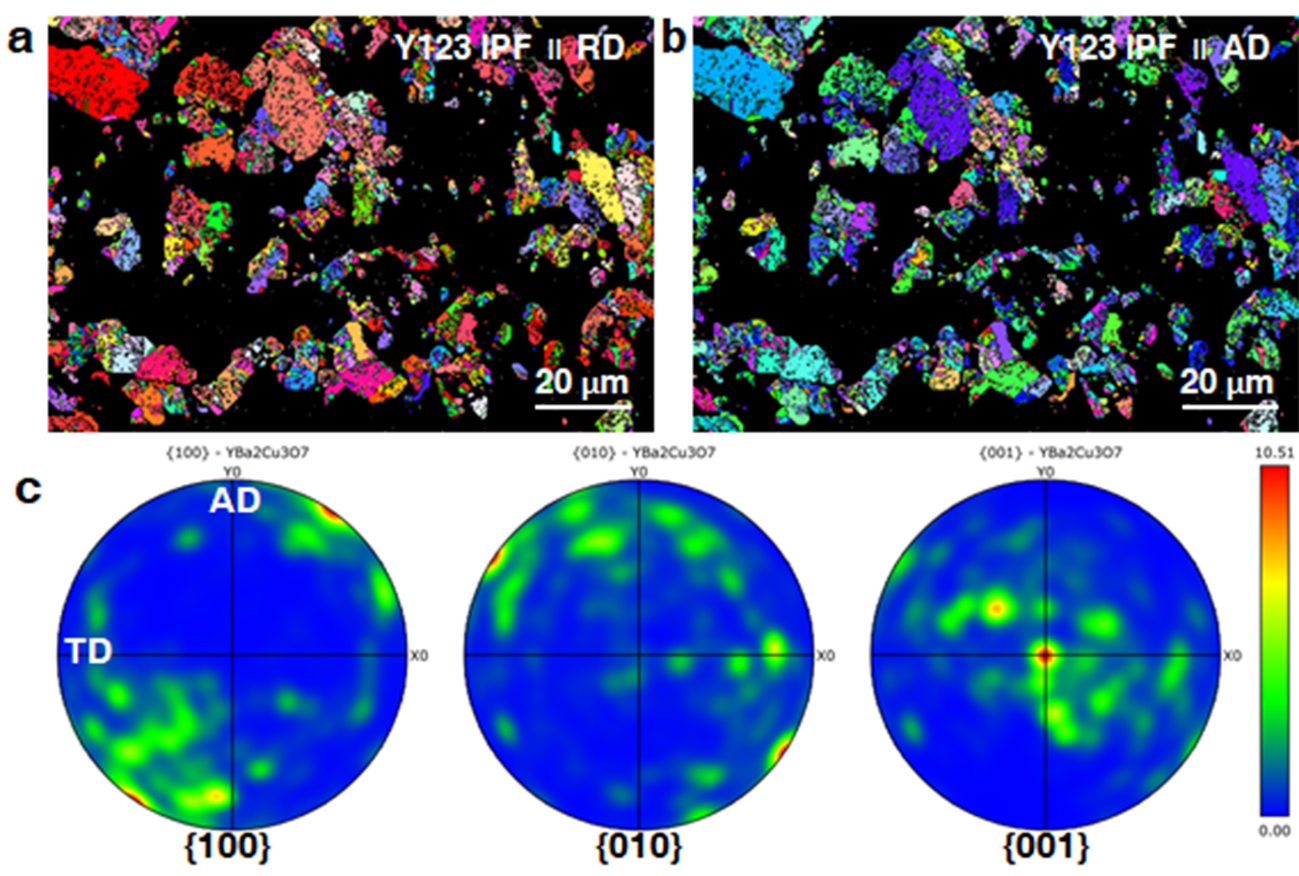


**Figure S15.** EBSD characterization of the small, nearly equiaxed pores structure formed by the fast-freezing condition. (a) IPF map along RD. (b) IPF map along AD. (c) PF of the {100}, {010}, and {001} crystallographic planes.


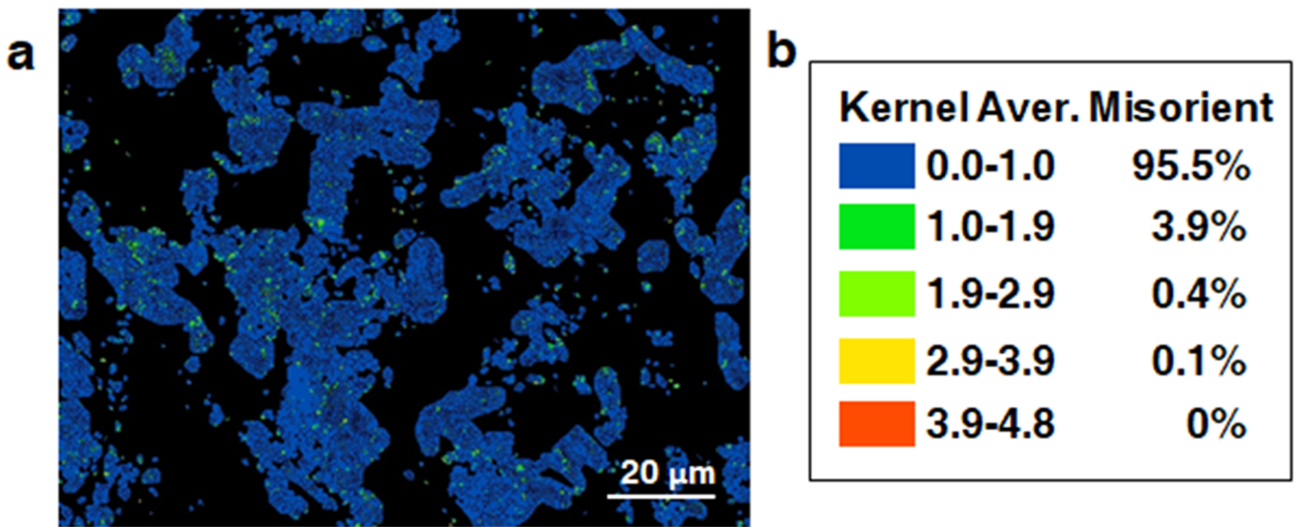


**Figure S16.** KAM map of Ag-YBCO composite. (a) KAM distribution map. (b) Color legend.


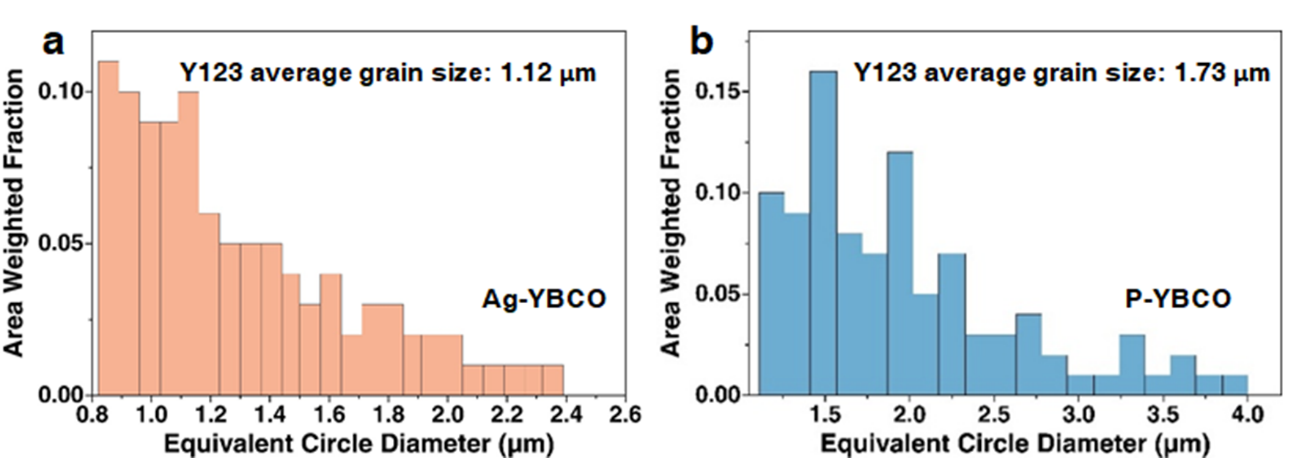


**Figure S17.** Grain size analysis. (a) Grain size of the Y123 phase in Ag–YBCO. (b) Grain size of the Y123 phase in P-YBCO.


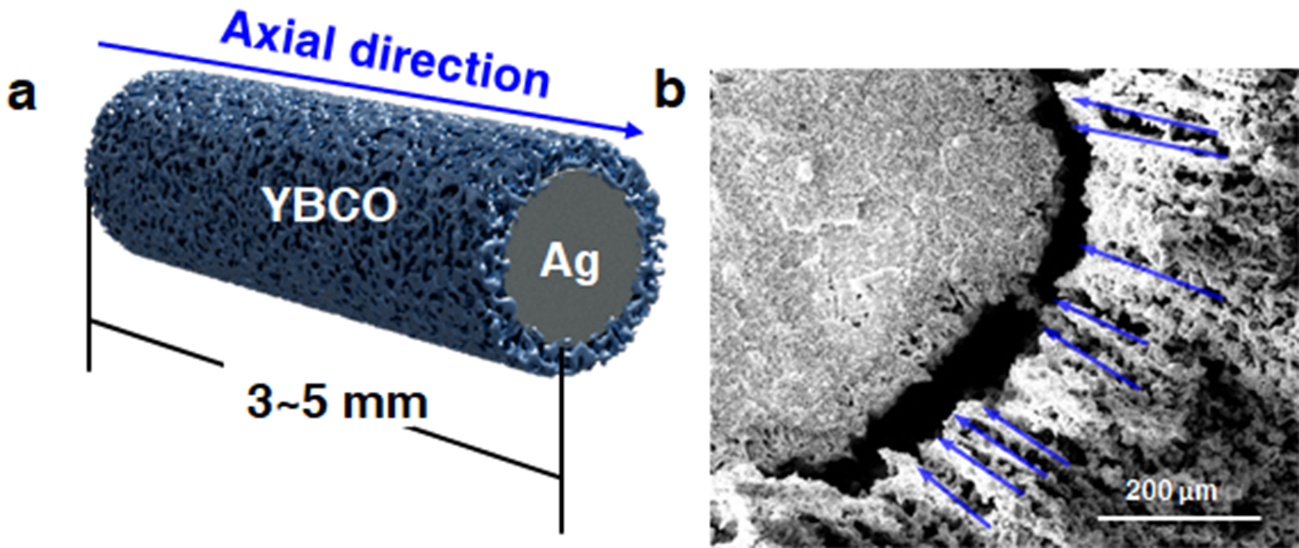


**Figure S18.** Fabrication of 3~5 mm Ag–YBCO cylindrical short samples using dual-material nozzle co-extrusion. (a) Schematic of the short sample. (b) Radial pores formed after freeze-drying.


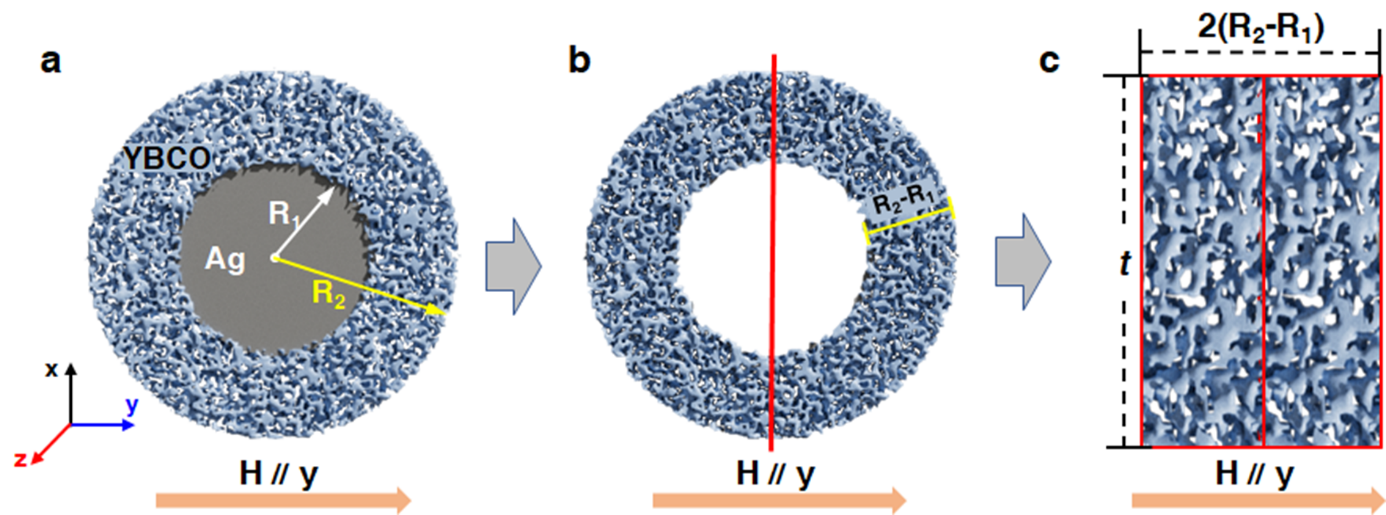


**Figure S19.** Extended Bean model for Ag-YBCO composite structure. (a) Cross-section of the short sample. (b) YBCO shell cross-section. (c) Geometrically equivalent model of two superimposed strips.


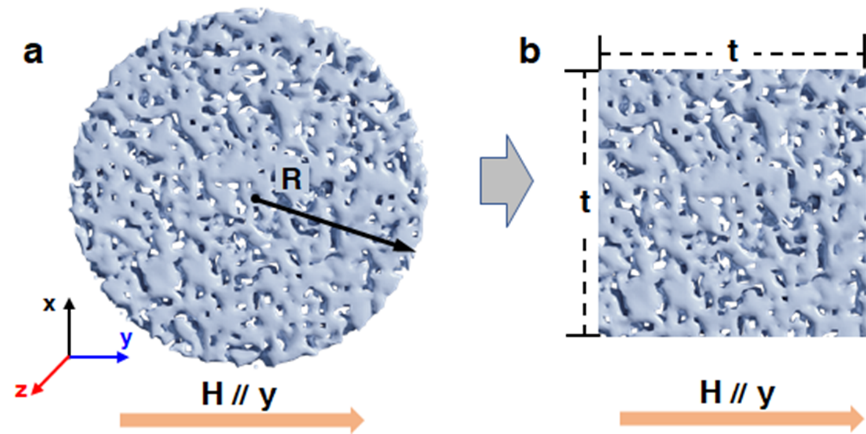


**Figure S20.** Extended Bean model for P-YBCO. (a) Cross-section. (b) Equivalent model.


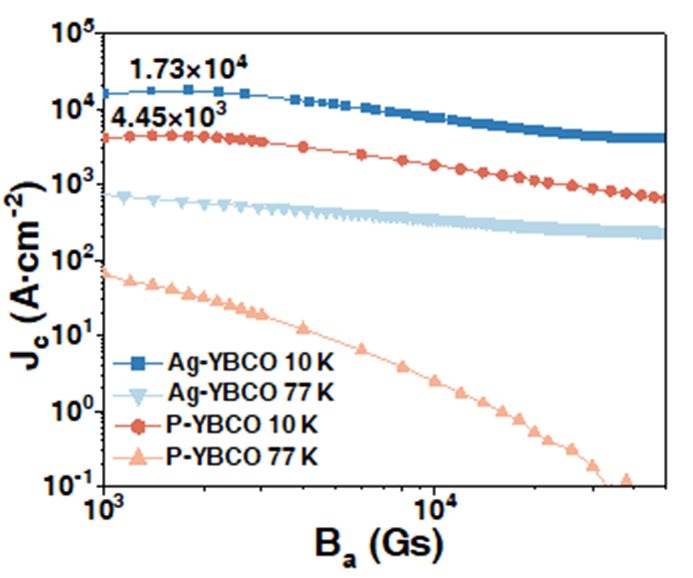


**Figure S21.** *J*_c_ of Ag-YBCO and P-YBCO at different temperatures calculated using the extended Bean model.


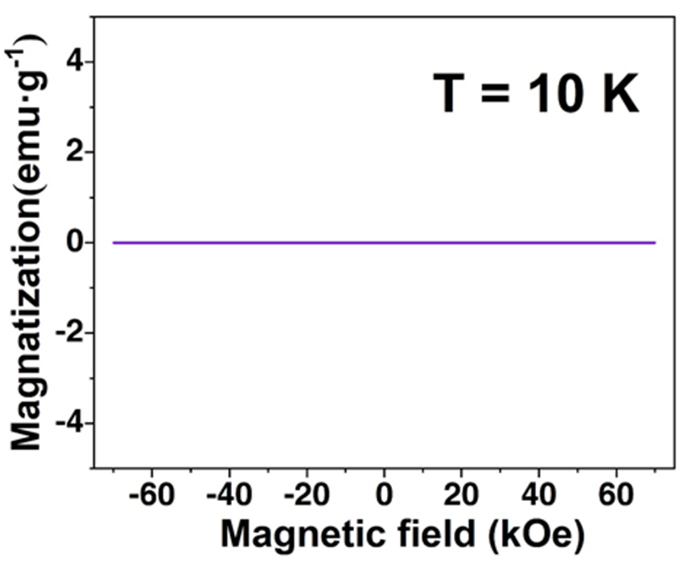


**Figure S22.** *M-H* curve of pure Ag at 10 K.


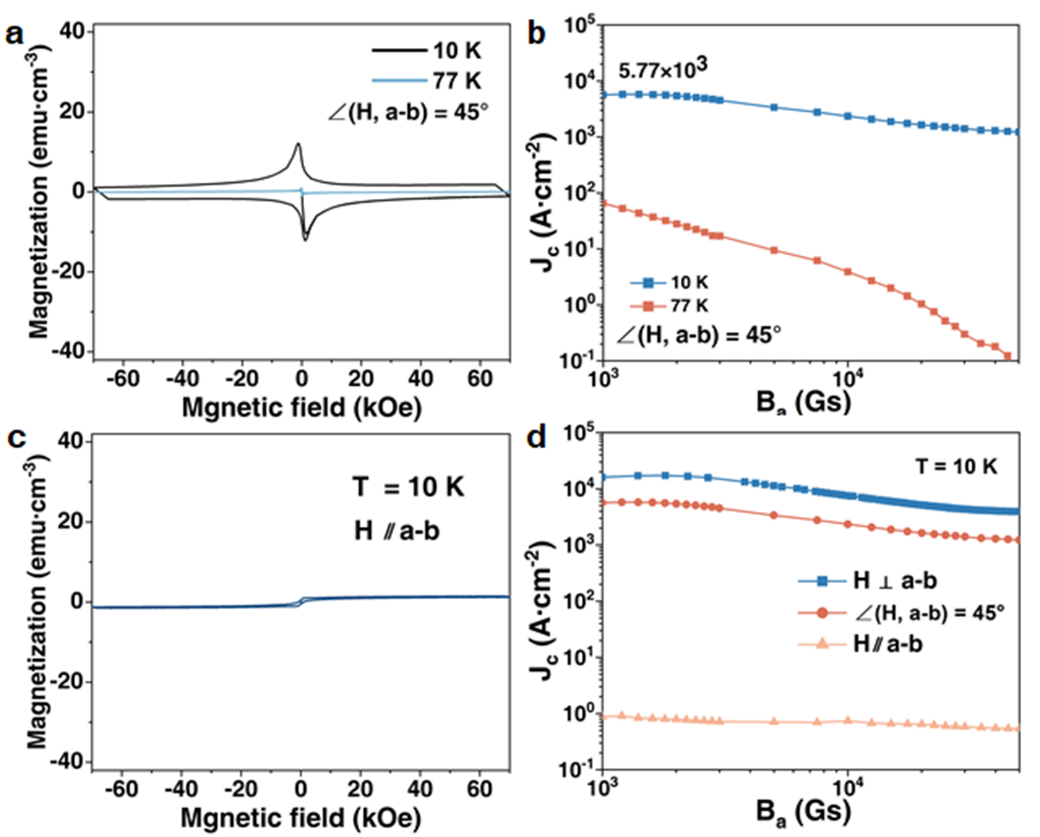


**Figure S23.** *M-H* curves of the Ag-YBCO composite with H ∥ ab.


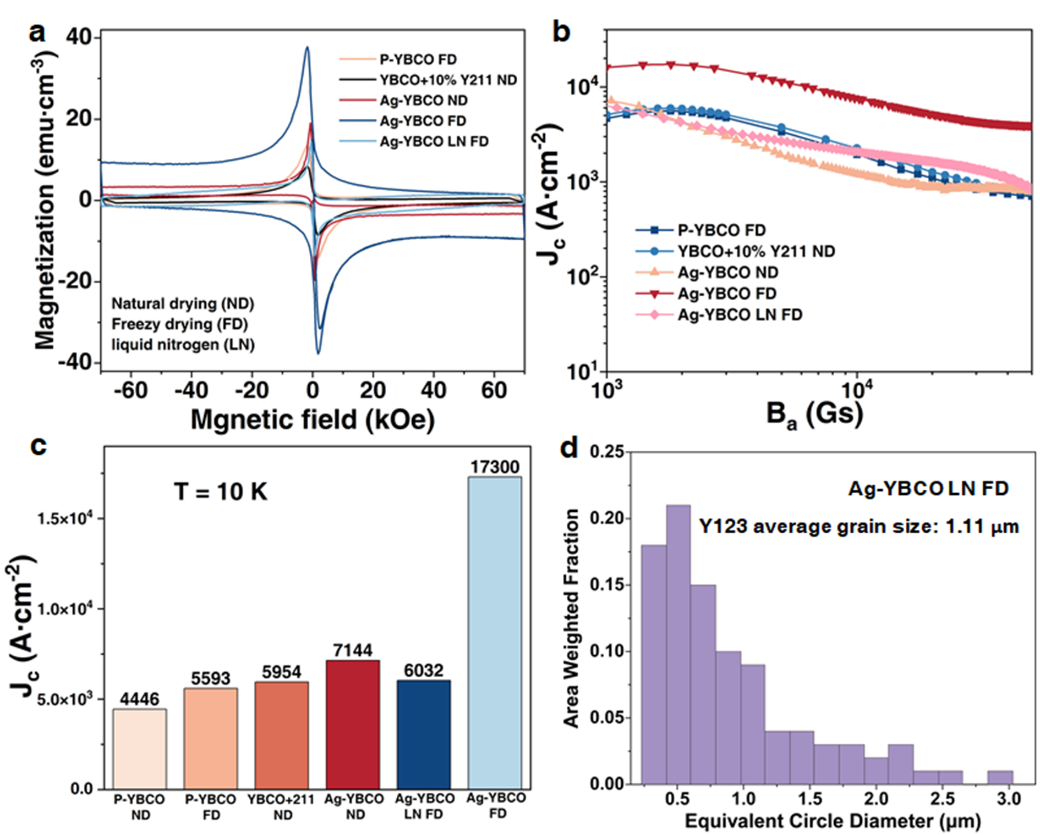


**Figure S24.** Quantitative assessment of the individual and synergistic contributions of microstructural factors to the *J*_c_ in YBCO-based samples.) (a) *M–H* curves measured at 10 K for a series of control samples, including naturally dried pure YBCO (P-YBCO ND, reference), freeze-dried pure YBCO (P-YBCO FD), naturally dried YBCO with 10 wt.% Y211 addition (YBCO + 10% Y211 ND), naturally dried Ag-YBCO (Ag-YBCO ND), liquid-nitrogen-quenched Ag-YBCO prepared via freeze drying (Ag-YBCO LN FD), and freeze-dried Ag-YBCO fabricated under the optimized process (Ag-YBCO FD). (b,c) Corresponding *J*_c_ values extracted from the *M–H* loops, enabling quantitative comparison of the effects of pore formation, Y211 flux pinning, Ag-induced grain-boundary bridging, and grain refinement on current-carrying performance. (d) Representative microstructural characterization showing grain refinement in the Ag-YBCO LN FD sample, with an average grain size of ~1.11 μm, illustrating the limited contribution of grain refinement alone in the absence of optimized pore connectivity and Ag distribution.


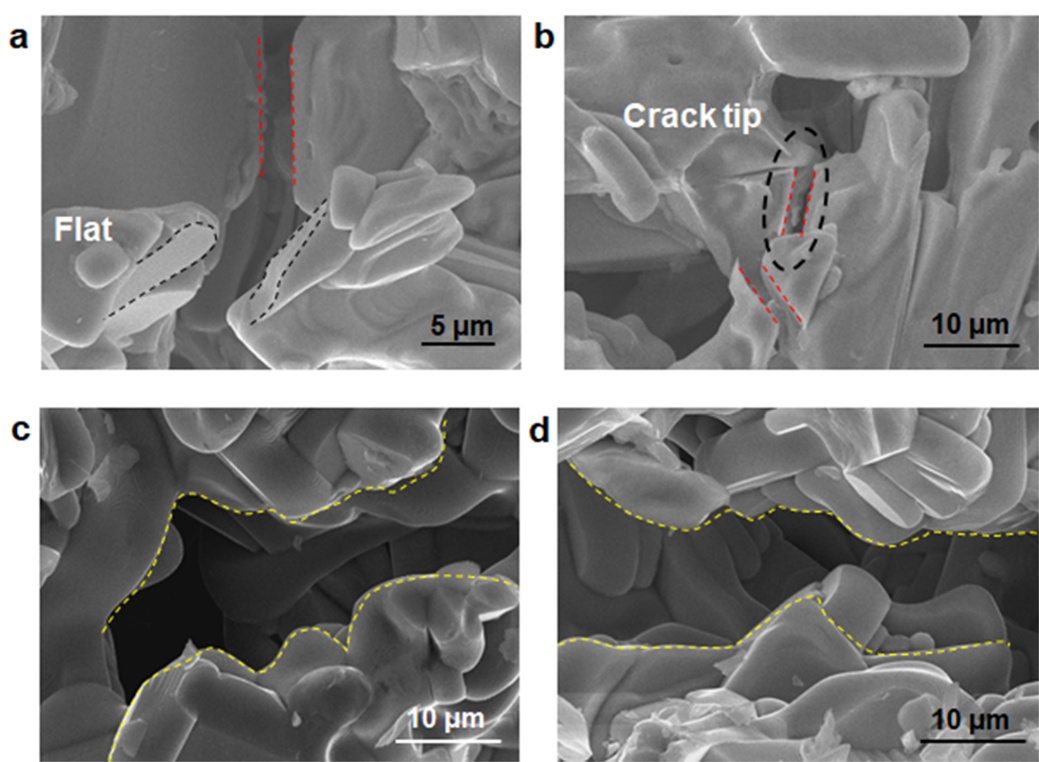


**Figure S25.** SEM images of the fracture surfaces. (a,b) SEM micrographs of an Ag-YBCO sample exhibiting transgranular fracture. (c,d) SEM micrographs of a P-YBCO sample showing intergranular fracture.


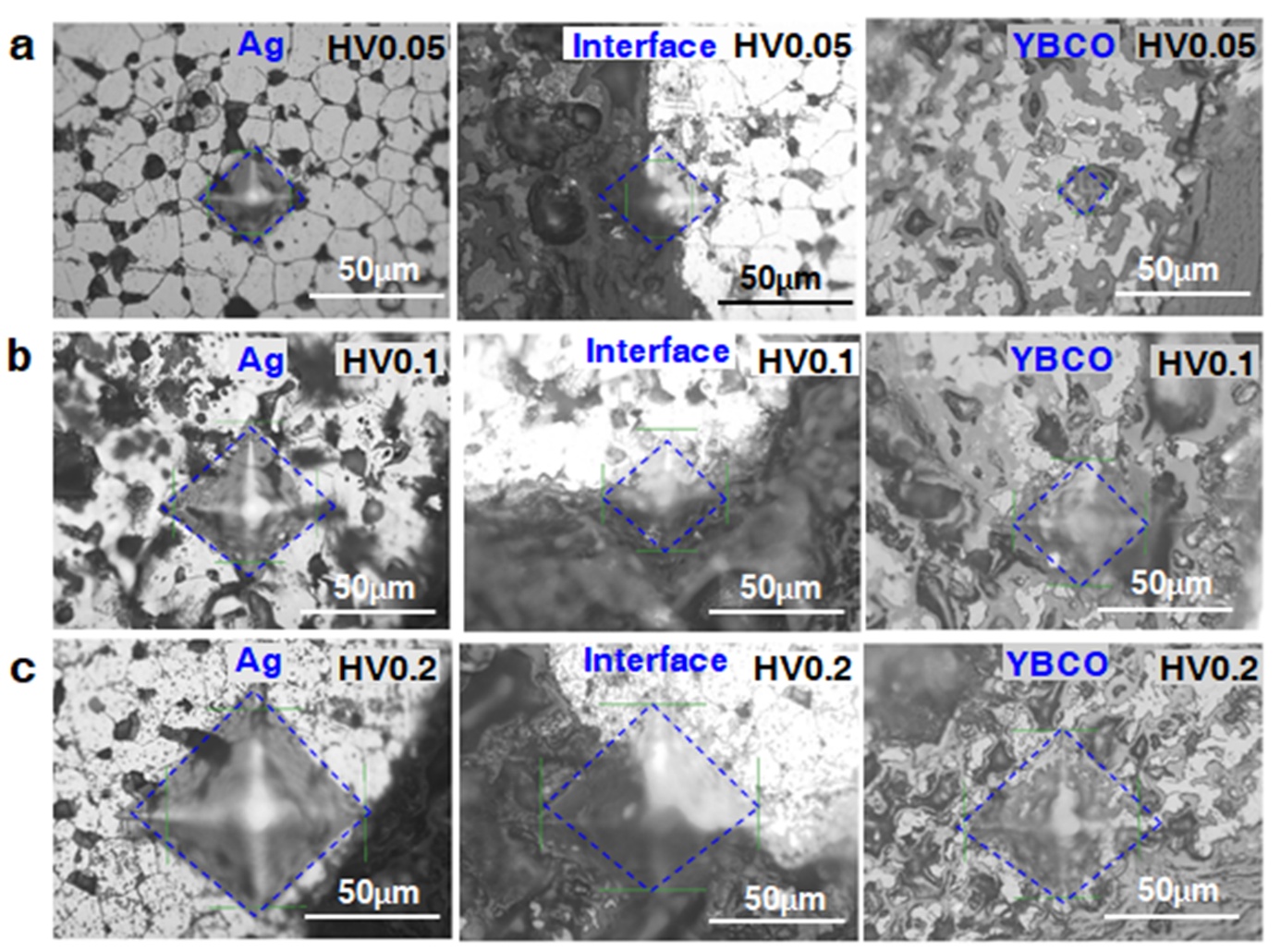


**Figure S26.** Micro-Vickers hardness indentation morphologies. (a) 0.05 kgf load (HV 0.05). (b) 0.10 kgf load (HV 0.10). (c) 0.20 kgf load (HV 0.20).


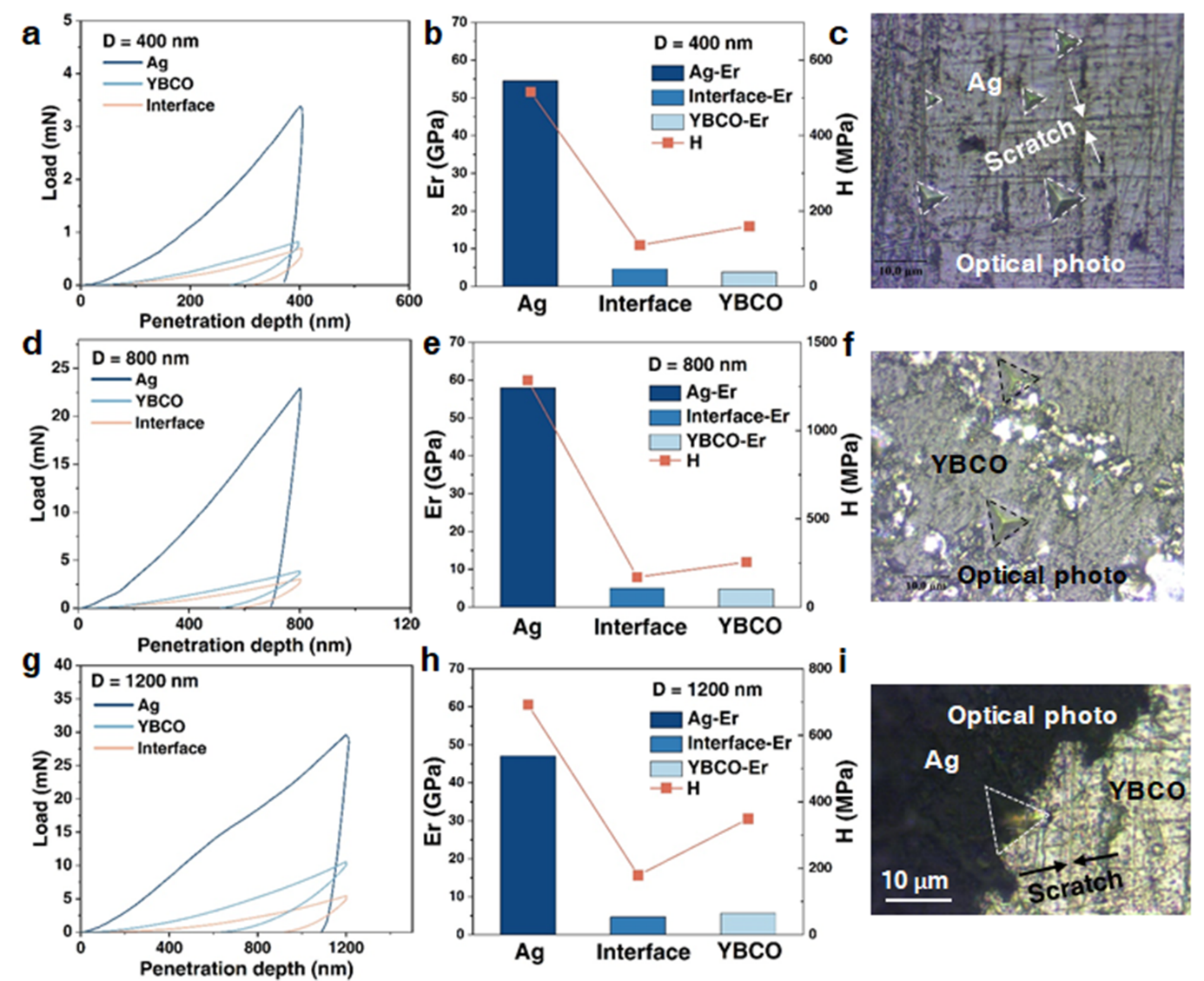


**Figure S27.** Nanoindentation load-displacement curves and morphology. (a,b) D=400 nm. (c) Indent in the Ag region. (d,e) D=800 nm. f, Indent in the YBCO region. (g,h) D=1200 nm. (i) Indent at the interface.


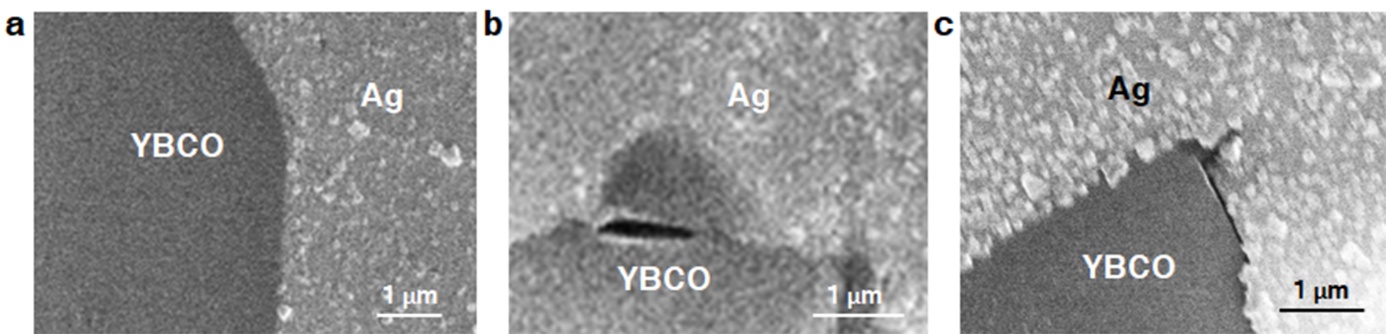


**Figure S28.** High-resolution SEM characterization of the Ag–YBCO bonded interface. (a) The Ag-YBCO interface is generally dense and well bonded, with no discernible macroscopic pores or interfacial cracks. (b,c) Careful SEM observations reveal the presence of sparse, locally distributed nanoscale pores at the Ag–YBCO interface, appearing as elongated features along the interfacial region.

**Table S1.** Possible phases and their crystal structures in the YBCO-Ag system.

| **Possible phases in the system** | **Crystal structure** | **Lattice constant** | | |
| --- | --- | --- | --- | --- |
|  |  | ***a*(Å)** | ***b*(Å)** | ***c*(Å)** |
| YBa_2_Cu_3_O_7_(Y123)^8^ | Orthorhombic | 3.816 | 3.882 | 11.674 |
| Y_2_BaCuO_5_(Y211)^9^ | Orthorhombic | 5.6593 | 7.1319 | 12.1802 |
| YBa_2_Cu_3-x_Ag_x_O_7-δ_(YBCAO)^10^ | Orthorhombic | 3.8308 | 3.8933 | 11.7170 |
| Ag^11^ | Cubic | 4.0790 | 4.0790 | 4.0790 |
| Ag_2_O^12^ | Cubic | 4.7200 | 4.7200 | 4.7200 |

**Table S2.** Phase composition and elemental distribution analysis by EPMA.

| **Point** | **EPMA results（at.%）** | | | | | **Phase** |
| --- | --- | --- | --- | --- | --- | --- |
|  | **Y** | **Ba** | **Ag** | **Cu** | **O** |  |
| P1 | 7.7554 | 15.0966 | 1.0740 | 22.2534 | 53.8206 | YBCAO(x≈0.14) |
| P2 | 7.8441 | 14.9218 | 1.4878 | 22.3134 | 53.4330 | YBCAO(x≈0.19) |
| P3 | 7.4833 | 14.5341 | 1.3155 | 22.4297 | 54.2374 | YBCAO(x≈0.17) |
| P4 | 8.0162 | 14.0159 | 0 | 23.1781 | 54.7897 | Y123 |
| P5 | 8.1256 | 14.2144 | 0 | 23.5751 | 54.0849 | Y123 |
| P6 | 8.4552 | 14.3539 | 0 | 24.3137 | 52.8772 | Y123 |

**Table S3.** Detailed lattice parameters of Ag-doped Yba_2_Cu_3_O_7−δ_ supercell models before and after structural optimization.

| **Phase** | **Supercell** | **Structure** | **Cell volume (Å^3^)** | **Density (g/cm^3^)** | **Lattice parameters** | | |
| --- | --- | --- | --- | --- | --- | --- | --- |
|  |  |  |  |  | ***a*(Å)** | ***b*(Å)** | ***c*(Å)** |
|  |  |  |  |  | **α** | **β** | **γ** |
| YBa_2_Cu_3_O_7_ | 2×3×1 | Orthorhombic | 1037.30 | 6.3988 | 7.6894 | 11.7786 | 11.6637 |
|  |  |  |  |  | 90° | 90° | 90° |
| YBaCuO_6.5_  (Y_2_Ba_4_Cu_6_O_13_) | 3×1×1 | Orthorhombic | 1048.32 | 6.2555 | 11.7175 | 7.7413 | 11.7721 |
|  |  |  |  |  | 90° | 90° | 90° |
| YBa_2_Cu_3_O_6_ | 2×3×1 | Tetragonal | 1101.01 | 5.9506 | 7.7812 | 11.6717 | 11.8230 |
|  |  |  |  |  | 90° | 90° | 90° |
| YBa_2_Cu_3_O_6.8_  (Y_5_Ba_10_Cu_15_O_34_) | 2×2×1 | Triclinic | 3545.15 | 6.2732 | 17.2239 | 17.5588 | 12.3660 |
|  |  |  |  |  | 106.59° | 98.41° | 88.52° |
| YBa_2_Cu_3_O_6.75_  (Y_4_Ba_8_(Cu_4_O_9_)_3_) | 2×2×1 | Monoclinic | 2848.91 | 6.0564 | 15.2626 | 15.7395 | 12.4507 |
|  |  |  |  |  | 90° | 107.73° | 90° |
| **After geometry optimization** | | | | | | | |
| YBa_2_Cu_2.8333_Ag_0.1667_O_7_  (Y_6_Ba_12_Cu_17_AgO_42_) | 2×3×1 | Orthorhombic | 1040.86 | 6.4476 | 7.6949 | 11.7694 | 11.7286 |
|  |  |  |  |  | 90° | 90° | 90° |
| YBa_2_Cu_2.8333_Ag_0.1667_O_6.5_  (Y_6_Ba_12_Cu_17_AgO_39_) | 1×3×1 | Orthorhombic | 1084.00 | 6.1175 | 11.7275 | 7.7206 | 11.8820 |
|  |  |  |  |  | 88.41° | 89.87° | 90.01° |
| YBa_2_Cu_2.8333_Ag_0.1667_O_6_  (Y_6_Ba_12_Cu_17_AgO_36_) | 2×3×1 | Tetragonal | 1115.01 | 6.1306 | 7.7754 | 11.6821 | 11.9471 |
|  |  |  |  |  | 88.71° | 91.18° | 90.05° |
| YBa_2_Cu_2.85_Ag_0.15_O_6.8_  (Y_20_Ba_40_Cu_57_Ag_3_O_136_) | 2×2×1 | Triclinic | 3614.88 | 6.1522 | 17.3059 | 17.5675 | 12.5297 |
|  |  |  |  |  | 106.44° | 98.26° | 88.87° |
| YBa_2_Cu_2.8125_Ag_0.1875_O_6.75_  (Y_16_Ba_32_Cu_45_Ag_3_O_108_) | 2×2×1 | Monoclinic | 2904.96 | 6.1324 | 15.4329 | 15.7868 | 12.5318 |
|  |  |  |  |  | 90° | 107.93° | 90° |

**Table S4.** Atomic displacements of O(1) and O(2) and Ag–O bond-angle deviations in optimized Ag-doped Yba_2_Cu_3_O_7−δ_ supercell models.

| **Crystal cell**  **configuration** | **Atomic coordinates** | **Atomic displacements** | | **Bond-angle variations** | | | |
| --- | --- | --- | --- | --- | --- | --- | --- |
|  | **O(1)(x,y,z)** | **O(2)(x,y,z)** | **O(1)(Å)** | | **O(2)(Å)** | **Ag-O(1)** | **Ag-O(2)** |
| YBa_2_Cu_3_O_7_ | (7.6249,0,1.9974) | (7.6249,2.0202,0) | 0.1321 | | 0.0861 | 0.93° | 0.52° |
| YBa_2_Cu_2.8333_Ag_0.1667_O_7_ | (7.6894,0,1.8821) | (7.6894,1.9631,0) |  |  |  |  |  |
| YBa_2_Cu_3_O_6.5_ | (11.7275,7.7206,1.8969) | (9.7729,7.7206,0) | 0.1422 | | 0.0914 | 0.57° | 0.59° |
| YBa_2_Cu_2.8333_Ag_0.1667_O_6.5_ | (11.7097,7.6696,2.0284) | (9.6970,7.6696,0) |  |  |  |  |  |
| YBa_2_Cu_3_O_6_ | (7.6931,0,1.9960) | / | 0.2054 | | / | 1.45° | / |
| YBa_2_Cu_2.8333_Ag_0.1667_O_6_ | (7.7812,0,1.8105) | / |  |  |  |  |  |
| YBa_2_Cu_3_O_6.8_ | (2.2652,0.1660, 9.7773) | (3.3975,2.6355, 11.0754) | 0.3430 | | 0.2363 | 0.53° | 0.82° |
| YBa_2_Cu_2.85_Ag_0.15_O_6.8_ | (2.3022,0.2593, 10.1053) | (3.4218,2.5073, 11.2724) |  |  |  |  |  |
| YBa_2_Cu_3_O_6.75_ | (3.0305,1.9650, 9.3115) | (3.6950,4.0827, 11.2718) | 0.2409 | | 0.1511 | 0.24° | 0.69° |
| YBa_2_Cu_2.8125_Ag_0.1875_O_6.75_ | (3.0886,1.9734, 9.5451) | (3.6924,3.9467, 11.3375) |  |  |  |  |  |

**Table S5.** Bulk energies (*E*_bulk_) and surface slab energies (*E*_slab_) of optimized Ag-doped Yba_2_Cu_3_O_7−δ_ supercell models.

| **Phase** | **E_bulk_ (ev)** | **Surface** | **E_slab_ (ev)** | **n_slab_** | **Surface energies (J·m^-2^)** |
| --- | --- | --- | --- | --- | --- |
| YBa_2_Cu_2.8333_Ag_0.1667_O_7_ | -2.1704×10^4^ | 100 | -8.6705×10^4^ | 4 | 6.4410 |
|  |  | 010 | -8.6731×10^4^ |  | 7.5245 |
|  |  | 001 | -8.6792×10^4^ |  | 2.1172 |
| YBa_2_Cu_2.8333_Ag_0.1667_O_6.5_ | -2.2382×10^4^ | 100 | -8.9441×10^4^ | 4 | 7.5394 |
|  |  | 010 | -8.9409×10^4^ |  | 6.7890 |
|  |  | 001 | -8.9486×10^4^ |  | 2.3961 |
| YBa_2_Cu_2.8333_Ag_0.1667_O_6_ | -2.2195×10^4^ | 100 | -8.8659×10^4^ | 4 | 6.9444 |
|  |  | 010 | -8.8707×10^4^ |  | 6.2946 |
|  |  | 001 | -8.8744×10^4^ |  | 3.1746 |
| YBa_2_Cu_2.85_Ag_0.15_O_6.8_ | -1.7873×10^5^ | 100 | -2.4616×10^6^ | 14 | 5.7597 |
|  |  | 010 | -2.4532×10^6^ |  | 7.0558 |
|  |  | 001 | -2.4725×10^6^ |  | 3.0511 |
| YBa_2_Cu_2.8125_Ag_0.1875_O_6.75_ | -2.0236×10^5^ | 100 | -2.3889×10^6^ | 12 | 6.2189 |
|  |  | 010 | -2.3891×10^6^ |  | 6.3293 |
|  |  | 001 | -2.3984×10^6^ |  | 3.8329 |

**Table S6.** Freeze–drying process parameters for pore structure control

| **Pore regime** | **Freezing protocol** | **Drying protocol** |
| --- | --- | --- |
| Large pores  (> 20 μm) | Controlled cooling at −2 °C·min^-1^ to −40°C, followed by isothermal holding for 2 h | Primary drying at −30°C for 12 h; secondary drying at 20°C for 8 h |
| Medium pores  (5–10 μm, optimal) | Accelerated cooling at −5°C·min^-1^ to −50°C, followed by isothermal holding for 1 h | Primary drying at −35°C for 24 h; secondary drying at 25°C for 8 h |
| Small pores  (< 5 μm, irregular) | Rapid quenching in liquid nitrogen, followed by equilibration at −80°C for 0.5 h | Primary drying at −40°C for 36 h; secondary drying at 30°C for 10 h |

**Table S7.** Micro-Vickers hardness indentation data of different regions in Ag-YBCO composite.

| **Test location** | **Load (Kg)** | **Hardness (MPa)** | **Indentation length**  ***d*_1_ (μm)** | **Indentation length**  ***d*_2_ (μm)** |
| --- | --- | --- | --- | --- |
| Ag | HV0.05 | 77.9 | 34.38 | 34.64 |
| Interface | HV0.05 | 110 | 29.13 | 29.01 |
| YBCO | HV0.05 | 250 | 19.60 | 18.91 |
| Ag | HV0.1 | 52.5 | 60.02 | 58.84 |
| Interface | HV0.1 | 60.0 | 56.30 | 54.89 |
| YBCO | HV0.1 | 63.4 | 55.23 | 52.96 |
| Ag | HV0.2 | 54.4 | 82.54 | 82.60 |
| Interface | HV0.2 | 67.4 | 75.18 | 73.19 |
| YBCO | HV0.2 | 72.8 | 72.55 | 70.16 |

**References**

1 Min Cheong, C. & Kien Chen, S. First principle calculation of electronic structures and hole concentration of YBCO family compounds. *Materials Today: Proceedings* **96**, 94-99, doi:https://doi.org/10.1016/j.matpr.2023.11.089 (2024).

2 Lopez, G. M., Filippetti, A., Mantega, M. & Fiorentini, V. First-principles calculation of electronic and structural properties of YBa_2_Cu_3_O_6+y_. *Physical Review B* **82**, 195122, doi:10.1103/PhysRevB.82.195122 (2010).

3 Parmigiani, F., Samoggia, G., Calandra, C. & Manghi, F. Surface stoichiometry and valence electronic structure of YBa_2_Cu_3_O_7−x_. *Journal of Applied Physics* **66**, 5958-5961, doi:10.1063/1.343623 (1989).

4 Islam, Z. *et al.* Four-Unit-Cell Superstructure in the Optimally Doped YBa_2_Cu_3_O_6.92_ Superconductor. *Physical Review Letters* **93**, 157008, doi:10.1103/PhysRevLett.93.157008 (2004).

5 Wang, F. *et al.* Orientation Relationships and Interface Structure in MgAl_2_O_4_ and MgAlB_4_ Co-Reinforced Al Matrix Composites. *ACS Applied Materials & Interfaces* **11**, 42790-42800, doi:10.1021/acsami.9b14923 (2019).

6 Dholabhai, P. P. & Uberuaga, B. P. Beyond Coherent Oxide Heterostructures: Atomic-Scale Structure of Misfit Dislocations. *Advanced Theory and Simulations* **2**, 1900078, doi:https://doi.org/10.1002/adts.201900078 (2019).

7 Gyorgy, E. M., van Dover, R. B., Jackson, K. A., Schneemeyer, L. F. & Waszczak, J. V. Anisotropic critical currents in Ba_2_YCu_3_O_7_ analyzed using an extended Bean model. *Applied Physics Letters* **55**, 283-285, doi:10.1063/1.102387 (1989).

8 Voronin, V. I., Goshchitskii, B. N., Mitberg, É. B., Leonidov, I. A. & Kozhevnikov, V. L. Valence state of cobalt atoms and crystal structure of YBa_2_Cu_3−x_Cu_x_O_6+δ_ solid solution. *Journal of Structural Chemistry* **41**, 626-631, doi:10.1007/BF02683925 (2000).

9 Wong-Ng, W., McMurdie, H., Paretzkin, B., Hubbard, C. & Dragoo, A. ICDD Grant-in-Aid. *NBS, GAITHERSBURG, MD, USA* (1987).

10 Behera, D., Mishra, N. C. & Patnaik, K. Ag-doping-induced coordination incompatibility and its effect on superconductivity in YBCO. *Journal of Superconductivity* **10**, 27-32, doi:10.1007/BF02763947 (1997).

11 Davey, W. P. Precision Measurements of the Lattice Constants of Twelve Common Metals. *Physical Review* **25**, 753-761, doi:10.1103/PhysRev.25.753 (1925).

12 Hanawalt, J., Rinn, H. & Frevel, L. Chemical analysis by X-ray diffraction. *Industrial & Engineering Chemistry Analytical Edition* **10**, 457-512 (1938).
